# A Novel ASCT2 Inhibitor, C118P, Blocks Glutamine Transport and Exhibits Antitumour Efficacy in Breast Cancer

**DOI:** 10.3390/cancers15205082

**Published:** 2023-10-20

**Authors:** Xiao-Dan Lyu, Yang Liu, Jia Wang, Yuan-Cheng Wei, Yi Han, Xue Li, Qian Zhang, Zheng-Rui Liu, Zheng-Zheng Li, Jing-Wei Jiang, Hao-Lin Hu, Sheng-Tao Yuan, Li Sun

**Affiliations:** 1 Jiangsu Key Laboratory of Drug Screening, China Pharmaceutical University, Nanjing 210009, China; lyud12@163.com (X.-D.L.); liuyang@njucm.edu.cn (Y.L.); wjj07032022@163.com (J.W.); weiyuancheng2000@163.com (Y.-C.W.); hanhan9934@163.com (Y.H.); lixue1130279675@126.com (X.L.); 3219071101@stu.cpu.edu.cn (Q.Z.); ccyfshengwu@163.com (Z.-R.L.); 15151865760@163.com (Z.-Z.L.); 2 School of Medicine and Holistic Integrative Medicine, Nanjing University of Chinese Medicine, Nanjing 210023, China; 3 Shuangyun BioMed Sci & Tech Co., Ltd., Suzhou 215000, China; jiangjingwei@126.com; 4 General Surgery, Zhongda Hospital, School of Medicine, Southeast University, Nanjing 210009, China; huhaolin1234@126.com; 5 Jiangsu Center for Pharmacodynamics Research and Evaluation, China Pharmaceutical University, Nanjing 210009, China

**Keywords:** C118P, breast neoplasms, cell proliferation, ASCT2, interleukin-6

## Abstract

**Simple Summary:**

ASCT2 is an attractive tumour metabolism target based on its critical role in cancer cell growth. The potential mechanisms of microtubule protein inhibitor C118P in breast cancer remain unknown. Identification of the potential target of C118P is essential. We evaluated the inhibitory effects of C118P on breast cancer. C118P restrained the tumour growth of MDA-MB-231 cells by inducing apoptosis, G2/M phase arrest, and autophagy. Furthermore, ASCT2 was confirmed to be a target of C118P. This is the first report to provide evidence that ASCT2 might be a candidate target of C118P in breast cancer treatment. Remarkably, this study will provide an opportunity for ASCT2 inhibition as a therapeutic strategy.

**Abstract:**

Background: The microtubule protein inhibitor C118P shows excellent anti-breast cancer effects. However, the potential targets and mechanisms of C118P in breast cancer remain unknown. Methods: Real-time cellular analysis (RTCA) was used to detect cell viability. Apoptosis and the cell cycle were detected by flow cytometry. Computer docking simulations, surface plasmon resonance (SPR) technology, and microscale thermophoresis (MST) were conducted to study the interaction between C118P and alanine-serine-cysteine transporter 2 (ASCT2). Seahorse XF technology was used to measure the basal oxygen consumption rate (OCR). The effect of C118P in the adipose microenvironment was explored using a co-culture model of adipocytes and breast cancer cells and mouse cytokine chip. Results: C118P inhibited proliferation, potentiated apoptosis, and induced G2/M cell cycle arrest in breast cancer cells. Notably, ASCT2 was validated as a C118P target through reverse docking, SPR, and MST. C118P suppressed glutamine metabolism and mediated autophagy via ASCT2. Similar results were obtained in the adipocyte–breast cancer microenvironment. Adipose-derived interleukin-6 (IL-6) promoted the proliferation of breast cancer cells by enhancing glutamine metabolism via ASCT2. C118P inhibited the upregulation of ASCT2 by inhibiting the effect of IL-6 in co-cultures. Conclusion: C118P exerts an antitumour effect against breast cancer via the glutamine transporter ASCT2.

## 1. Introduction

Metabolic reprogramming, which fuels tumour cell growth, is considered an emerging hallmark of cancer [1]. Cancer cell metabolic remodelling is characterised by the aberrant metabolism of glucose, amino acids, and lipids [2]. In addition to their dependency on aerobic glycolysis, cancer cells exhibit other metabolic adaptations, such as increased fatty acid synthesis and “glutamine addiction” [3]. Glutamine metabolic reprogramming is mainly associated with several glutamine metabolic enzymes, such as glutaminase (GLS), glutamine synthetase (GLUL), and the glutamine transporter family (solute carrier (SLC) family). Among SLC family members, alanine-serine-cysteine transporter 2 (ASCT2, encoded by the *SLC1A5*) is recognised as the principal glutamine transporter and is critical for glutamine uptake in tumour cells. Compared with its expression in healthy tissues, ASCT2 is overexpressed in many tumours, such as non-small cell lung cancer (NSCLC) [4], breast cancer [5], and hepatocellular carcinoma [6]. Previous studies have mainly focused on the pro-proliferative effect of ASCT2 on tumours, such as breast cancer [7], prostate cancer [8], melanoma [9], NSCLC, colon cancer, and endometrial cancer [10]. A recent study revealed the critical role of ASCT2-mediated amino acid metabolism in promoting leukaemia development and progression [11]. Thus, these studies indicate the importance of ASCT2 in tumour progression.

Since glutamine plays a critical role in cancer cell growth, new therapies targeting glutamine metabolism have attracted attention. One agent targeting GLS, CB-839, is currently being evaluated in phase II clinical trials. However, a limitation of targeting GLS is that these treatments may induce RAS-independent activation of MAPK signalling [12]. In addition to agents targeting GLS, anti-ASCT2 agents have been developed as potential antitumour drugs and have shown promise. In addition to the drug GPNA [13], H. Charles Manning and his team discovered a small-molecule inhibitor of ASCT2 (V-9302) [14]. However, V-9302 may not be a specific inhibitor of ASCT2, as it also targets SNAT2 (sodium-coupled neutral amino acid transporter 2, SLC38A2) and LAT1 (large neutral amino acid transporter 1, SLC7A5) [15]. In addition to small-molecule inhibitors, ASCT2 monoclonal antibodies are currently under investigation, but they do not appear to show selectivity between patients with low and high ASCT2 expression, which will limit their successful application [16].

CAAs in the tumour microenvironment promote metabolic remodelling in breast cancer. However, our understanding of the interplay between breast cancer cells and adipocytes on glutamine metabolism is incomplete. Then, we focused on the effect of the adipocyte–breast cancer microenvironment on glutamine metabolism. 

Therefore, we report the identification of a potential target of C118P, a new class 1 drug for which a clinical phase I trial has been approved. We aimed to identify biomarkers of C118P and recruit the appropriate patients to test the efficacy of C118P, and identified a potential target of C118P, ASCT2, via reverse docking. In this study, blockade of ASCT2 with C118P resulted in attenuated cancer cell growth and proliferation, increased cell apoptosis, and G2/M cell cycle arrest, which collectively contributed to the antitumour response of C118P in vitro and in vivo. In summary, investigating the effect and mechanism of C118P on inhibiting the proliferation of breast cancer cells is expected to provide guidance for the treatment of breast cancer in the clinic and promote the development of new drugs targeting metabolism.

## 2. Materials and Methods

### 2.1. The Chemicals and Reagents

C118P was supplied by Sanhome Pharmaceutical Co., Ltd. (Nanjing, China). Taxol was purchased from APExBio (Houston, TX, USA). An apoptosis detection kit (Annexin V-PI staining kit) was purchased from Vazyme Biotech Co., Ltd. (Nanjing, China). A cell cycle detection kit was obtained from Beyotime Biotechnology (Shanghai, China).

### 2.2. Cell Culture

Human breast cancer cells (MDA-MB-231, MDA-MB-468, BT-549, MCF-7, T47D, and BT-474 cells) were obtained from the Shanghai Institute of Life Science at the Chinese Academy of Sciences. All cell lines were authenticated by short tandem repeat (STR) analysis. Cells were cultured in DF12 medium (Gibco, Grand Island, NE, USA) containing 10% foetal bovine serum (FBS, PAN Biotech, Aidenbach, Germany) supplemented with 100 U/mL penicillin and 100 μg/mL streptomycin in a humidified atmosphere (BB15 incubator, Thermo, Dreieich, Germany) with 5% CO_2_ at 37 °C.

### 2.3. Cell Viability Assay

The effects of C118P on breast cancer cells (MDA-MB-231, MDA-MB-468, BT-549, MCF-7, T47D, and BT-474 cells) were determined using the MTT assay. Cell suspensions were prepared, and 1800 cells of each type were seeded into a 96-well plate. After incubation for 24 h, the cells were treated with C118P for another 72 h. Subsequently, 20 μL of an MTT solution (0.5 mg/mL) was added and incubated for another 4 h, and the medium was replaced with 150 μL of DMSO to dissolve formazan precipitates. The absorbance at 570 nm was detected using a universal microplate reader (Infinite M100, Tecan, Stadt Crailsheim, Germany). Inhibition rates were calculated with the following formula: inhibition rate (%) = (1 − absorbance of the treated group/absorbance of the control group) × 100.

### 2.4. Real-Time Cellular Analysis (RTCA)

An xCELLigence system is a novel approach developed by Roche Applied Science (Penzberg, Germany) to investigate cell growth, adhesion, and morphology in real time in a label-independent manner. A change in impedance is recorded as the cell index, which indicates cell number, cellular attachment, and morphology. Cells were seeded at a density of 8000 cells (MDA-MB-231) or 10,000 cells (MDA-MB-468) per well, placed on a rotating plate and incubated for 30 min, and subsequently placed in the xCELLigence system, which was linked to a 37 °C incubator with a humidified atmosphere containing 5% CO_2_ [17]. After incubation for 24 h, cells were treated with C118P and observed for 144 h.

### 2.5. Colony Formation Assay

The effect of combination treatment on cell proliferation was detected with colony formation assays. A total of 2000 cells were seeded into a 6-well plate and incubated for 24 h. Subsequently, the cells were treated with 0.025, 0.05, and 0.1 μM C118P for 14 days. The cells were then fixed with 0.5% crystal violet and stained with 4% formaldehyde. Colonies were then counted macroscopically.

### 2.6. Apoptosis Detection and Cell Cycle Analysis

Cells were collected with EDTA-free trypsin and washed with ice-cold PBS. Subsequently, the cells were suspended in 500 μL of binding buffer and stained with 5 μL of PI and 5 μL of FITC-conjugated Annexin V for 15 min. Apoptotic cells were analysed with a FACSCalibur flow cytometer (BD Biosciences, San Jose, CA, USA).

The cell cycle distribution was detected by PI staining. Cells were collected and fixed in 75% ethanol overnight after drug treatment. Then, the cells were washed with ice-cold PBS once and stained with PI for 30 min at 37 °C. Cell cycle analysis was performed using a FACSCalibur flow cytometer (BD Biosciences).

### 2.7. Western Blotting

Western blotting was performed as described in a previous study [18]. The following antibodies purchased from Cell Signaling Technology (Danvers, MA, USA) were used: anti-m-TOR (Cat#2983, RRID: AB_2105622), anti-p-m-TOR (Cat#5536, RRID: AB_10691552), anti-p70S6K (Cat#9202, RRID: AB_331676), anti-p-p70S6K (Cat#9234, RRID: AB_2269803), anti-Bcl-2 (Cat#2872, RRID: AB_10693462), anti-MCL-1 (Cat#4572, RRID: AB_2281980), anti-Bcl-xl (Cat#2762, RRID: AB_10694844), anti-Cyclin B1 (Cat#4138, RRID: AB_2072132), anti-CDK1 (Cat#77055, RRID: 77055), anti-p-CDK1 (Tyr15) (Cat#4539, RRID: AB_331676), anti-LAMP-1 (Cat#9091, RRID: AB_2687579), anti-LC3I/II (Cat#4108, RRID: AB_2137703), anti-Beclin1 (Cat#3495, RRID: AB_1903911), anti-p62 (Cat#23214, RRID: AB_2798858), anti-GLS1 (Cat#88964, RRID: AB_2800133), anti-gp130 (Cat#3732, RRID: AB_2125953), anti-STAT3 (Cat#9139, RRID: AB_331757), anti-p-STAT3 (Cat#9145, RRID: AB_2491009), anti-ERK1/2 (Cat#4695, RRID: AB_390779), and anti-p-ERK1/2 (Cat#4370, RRID: AB_2315112) antibodies. Anti-ASCT2 (Cat#ab237704) antibodies were purchased from Abcam (Cambridge, UK). Anti-GLUL (Cat#A5437, RRID: AB_2863503), anti-GDH (Cat#A5176, RRID: AB_2863476), anti-β-actin (Cat#AC004, RRID: AB_2737399), and anti-GAPDH (Cat#AC002, RRID: AB_2736879) antibodies were purchased from ABclonal Technology (Wuhan, China). Anti-rabbit IgG conjugated to HRP (Cat#7074, RRID: AB_2099233) and anti-mouse IgG conjugated to HRP (Cat#7076, RRID: AB_330924) (Cell Signaling Technology) were used as secondary antibodies, and enhanced chemiluminescence reagent (Millipore) was used for detection after exposure in a Gel Doc 2000 image analyser (Bio-Rad, Hercules, CA, USA).

### 2.8. SPR Analysis of Recombinant Proteins

SPR measurements were performed using a Biacore T200 instrument (GE Healthcare, Chicago, IL, USA). The ASCT2 protein (SL5-H5149) was purchased from ACRO Biosystems. C118P at different concentrations (0.15625 μM to 10 μM) was run over the SPR instrument with a CM5 chip (GE, Chicago, IL, USA) using running buffer containing 1.8 mM KH_2_PO_4_, 10 mM Na_2_HPO_4_, 137 mM NaCl, 2.7 mM KCl, and 0.005% Tween-20 (pH 7.8). The binding and dissociation rates were measured at a flow rate of 25 μL/min. Ligand injection was performed over 1.5 min, followed by flow with ligand-free buffer to analyse dissociation for 2.5 min. Curves were corrected for nonspecific ligand binding by subtracting the signal obtained for the negative control flow cell. The equilibrium KD was derived from a simple 1:1 interaction model using Reichert data evaluation software (version 1.7.1).

### 2.9. Microscale Thermophoresis (MST) Analysis of Recombinant Proteins

The Monolith Protein Labeling Kit RED-NHS (L001) was purchased from NanoTemper Technologies (Watertown, MA, USA). For NT.115 NanoTemper measurements, an infrared (IR) laser beam coupled to a light path (i.e., fluorescence excitation and emission) with a dichroic mirror is focused into the fluid sample through the same optical element used for fluorescence imaging. The IR laser is absorbed by the aqueous solution in the capillary and locally heats the sample with a 1/e^2^ diameter of 25 μm. Up to 24 mW of laser power was used to heat the sample without damaging the biomolecules. Thermophoresis of the protein in the presence of C118P at varying concentrations (0.15625 μM to 10 μM) was analysed for 30 s. Measurements were performed at room temperature, and the S.D. was calculated from three independent experiments. Data were normalised to either ΔFnorm [‰] (10*(Fnorm (bound)—Fnorm (unbound))) or the bound fraction (ΔFnorm [‰]/amplitude).

### 2.10. Detection of ATP, Glutamine, Glucose, and Lactate Levels

After transduction or treatment with C118P at various concentrations (0.025, 0.05, 0.1 μM) for 48 h, ATP was detected with an ATP assay kit (Beyotime Biotechnology). Glutamine was detected with a glutamine assay kit (Sigma, St. Louis, MO, USA), glucose was detected with a glucose assay kit (Whitman Biotech, Nanjing, China), and lactate production was detected with a lactate production kit (Sunshine Biotechnology Ltd., Thatoom, Thailand).

### 2.11. Glutamine Uptake Assay

After pretreatment with C118P (0.025, 0.05, 0.1 μM) for 48 h, cells (1 × 10^5^ cells/well) were incubated with [^3^H]-L-glutamine (400 nM, PerkinElmer, Shelton, CT, USA) in MEM (Life) for 15 min at 37 °C in the presence or absence of inhibitor. The cells were collected, transferred to a 96-well plate harvester (PerkinElmer), and analysed using a liquid scintillation counter (PerkinElmer).

### 2.12. Oxygen Consumption Rate (OCR) Measurements

The OCR was measured using an XF96 analyser (Seahorse Bioscience, North Billerica, MA, USA). Cells were seeded in 96-well XF96 cell culture plates at a density of 20,000 cells/well. After incubation for 48 h, the cells were treated with C118P (0.025, 0.05, 0.1 μM). The media were then removed, and the wells were washed in XF-modified DMEM (Seahorse Bioscience) at pH 7.4 supplemented with 1 mM glutamine (glycolysis and mitochondrial stress tests), 2.5 mM glucose, 1 mM sodium pyruvate, 0.5 mM carnitine, and 1 mM palmitate in complex with 0.2 mM BSA (mitochondrial stress tests) and incubated for 1 h at 37 °C without CO_2_. The OCR was measured in the basal state (1 mM palmitate in complex with 0.2 mM BSA) or after the injection of 5 μM oligomycin, 1 μM 2-[2-[4-(trifluoromethoxy) phenyl] hydrazinylidene]-propanedinitrile (FCCP), and rotenone with antimycin A (both at 0.5 μM). After the Seahorse Bioscience experiments, the proteins were quantified to normalise the results.

### 2.13. MDC Staining and LysoTracker Red Staining

Monodansylcadaverin (MDC, KeyGEN BioTECH, Nanjing, China) is an eosinophilic stain that is commonly used as a specific stain to detect autophagosome formation. LysoTracker Red (Beyotime Biotechnology, Shanghai, China) is commonly used as a specific stain to detect lysosomes. After pretreatment with C118P (0.025, 0.05, or 0.1 μM) for 48 h, cells were incubated with MDC for 30 min or with LysoTracker Red for 60 min. Anti-fluorescent quenching tablets were used to seal the cells, and cells were photographed under a fluorescent microscope.

### 2.14. Lentivirus Transfection and Overexpression Studies

Small hairpin RNAs (shRNAs) against human *SLC1A5* and a negative control shRNA with the following sequences were purchased from GenePharma (Suzhou, China): LV3-*SLC1A5*#1: 5′-GCTTGGTAGTGTTTGCCATCG-3′; LV3-*SLC1A5*#2: 5′-GGATGTGGGTTTACTCTTTGC-3′; and LV3-NC: 5′-TTCTCCGAACGTGTCACGT-3′. The ASCT2 expression plasmid pcDNA3.1-Flag-*SLC1A5* was purchased from the Public Protein/Plasmid Library (Nanjing, China). Small interfering RNAs (siRNAs) against human *SLC1A5* and a negative control with the following sequences were purchased from GenePharma: si*SLC1A5*#1: sense 5′-GCCUUGGCAAGUACAUUCUTT-3′; antisense 5′-AGAAUGUACUUGCCAAGGCTT-3′; si*SLC1A5*#2: sense 5′-GUCGACCAUAUCUCCUUGATT-3′; antisense 5′-UCAAGGAGAUAUGGUCGACTT-3′; negative control: sense 5′-UUCUUCCGAACGUGUCACGUTT-3′; antisense 5′-ACGUGACACGUUCGGAGAATT-3′. Transfection was performed as described previously [19].

### 2.15. Adipose–Breast Cancer Cell Co-Culture Model

Breast cancer cells (2.0–3.0 × 10^5^) were seeded in the upper well of the Corning Transwell co-culture chambers (Corning, NY, USA). When the cells adhered, the induced mature adipocytes were seeded in the lower chamber. The supernatant from the upper and lower chambers was replaced with DMEM/F12 containing 2% FBS. Subsequent experiments were performed after three days of co-culture.

### 2.16. Three-Dimensional Culture

Breast cancer cells were cultured in 3.8 mL of complete medium, 1 mL of methylcellulose solution, and 50 μL of the Matrigel gel (Corning) mixture. Obvious white dots were visualised at the bottom of the well after a 72 h incubation in the cell incubator. Next, 96-well plates were spread with 50 μL of Matrigel, and 200 mL of complete medium was added to each well. Single-cell spheres were seeded in 96-well plates. Observation and photos were recorded for 0 h and recorded for 7 consecutive days.

### 2.17. Detection of the Mouse Microarray

The sandwich antibody chip is a chip based on the Raybiotech sandwich (Peachtree Corners, GA, USA), which is a detection mode using two antibodies. The experiments were performed by drying, sealing, and incubating the samples, followed by an analysis of fluorescence.

### 2.18. Nude Mouse Xenograft Study

Female BALB/c athymic nude mice (5–6 weeks) with body weights from 18 to 22 g were purchased from the Model Animal Research Center of Nanjing University (Nanjing, China). A total of 2 × 10^6^ MDA-MB-231 cells transfected with shControl or sh*SLC1A5*#1 were injected into the subcutaneous tissue of the armpit. Tumours were grown until their volume reached 300 to 500 mm^3^, resected, and cut into small pieces. Subsequently, the tissue pieces were subcutaneously implanted into each of the nude mice. The mice were randomly divided into groups of six individuals each. C118P was administered by tail vein injection at a concentration of 50 mg/kg. The negative group was given an equal amount of normal saline. At 21 days after administration, the mice were euthanised, and the tumour tissues were then resected and assessed. Tumour volume (TV) was calculated by the following formula: TV (mm^3^) = A/2 × B^2^, where A represents the longest diameter of the tumour, and B represents the shortest diameter. Relative tumour volume (RTV) was calculated with the following formula: RTV = V_t_/V_0_, where V_t_ represents the TV on day t, and V_0_ represents the TV on day 0. The animal care and surgical procedures were guided by the Animal Care and Control Committee of China Pharmaceutical University.

### 2.19. Targeted Metabolomics Analysis

Metabolomics studies of mouse tumour tissue samples from different experimental groups were performed using LC-MS as the analytical method. Experiments were conducted by collecting biological samples, detecting samples with the instrument, and analysing the data, as previously described [20].

### 2.20. Plasmids and ASCT2 Expression and Purification

The *SLC1A5* was cloned into the pET-28b (GenScript, Nanjing, China) expression plasmid to produce recombinant ASCT2 with a histidine tag. E. coli strain BL21 (DE3) obtained from Tiangen Biotech Co., Ltd. (Beijing, China) was transformed with the plasmid and cultured on a selective antibiotic LB agar plate. After 16 h, a single colony was picked and cultured in 10 mL of LB medium containing 50 μg/mL kanamycin with vigorous shaking at 37 °C for 10 h. Then, 10 mL cultures were added to 250 mL of medium and cultured for 2 h. Next, protein expression was induced by the addition of IPTG to a final concentration of 0.5 mM. The cells were left to grow overnight at 16 °C and then harvested by centrifugation. Protein extraction and purification were performed using a Ni-NTA Fast Start Kit (QIAGEN, Hilden, Germany) and an AKTA system, respectively. Then, the purified protein was concentrated by centrifugal filter devices (Millipore, Burlington, MA, USA), mixed with glycerol to a final concentration of 20%, and stored at −80 °C until use.

### 2.21. Gene Expression Analysis

The GEPIA2 (Gene expression profiling interactive analysis, version 2) web server (http://gepia2.cancer-pku.cn/#analysis, accessed on 20 July 2022) was used to determine the difference in ASCT2 expression between the breast cancer tissues and the corresponding normal tissues from the TCGA database [21]. Violin plots of ASCT2 expression in different pathological stages of breast cancer were constructed using GEPIA2. The Human Protein Atlas (https://www.proteinatlas.org, accessed on 20 July 2022) was used to obtain the expression of ASCT2 protein in human tissues [22]. GEPIA2 was used to determine the significance of the association of OS (overall survival) with ASCT2 expression in breast cancer.

### 2.22. Statistical Analyses

All data in this study are expressed as the mean ± S.D. and were analysed using Student’s *t*-test (* *p* < 0.05, ** *p* < 0.01, *** *p* < 0.001, and N.S. represents no significant change).

## 3. Results

### 3.1. C118P Potently Inhibited the Proliferation of Breast Cancer Cell Lines In Vitro

**Figure 1:**
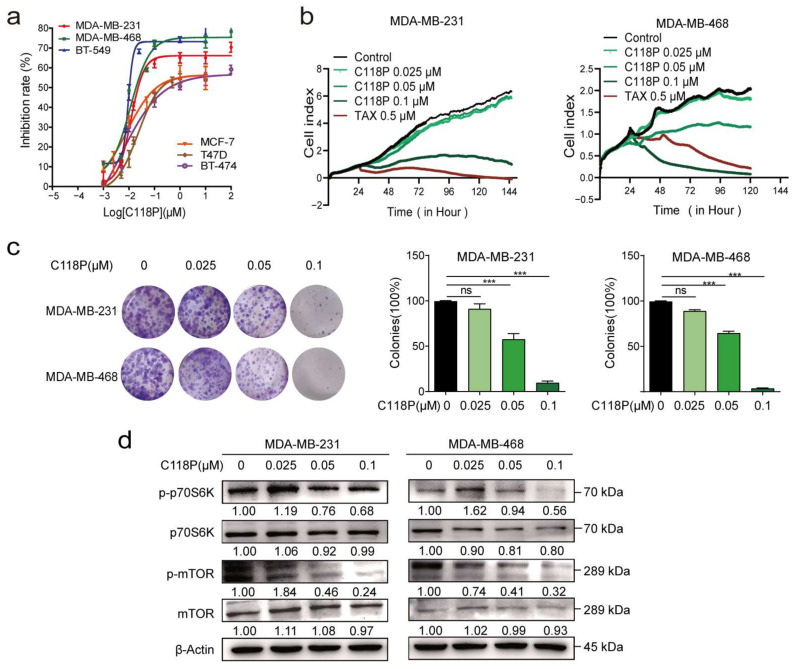
C118P potently inhibited the proliferation of breast cancer cells in vitro. Six breast cancer cell lines (MDA-MB-231, MDA-MB-468, BT-549, MCF-7, T47D, and BT-474) were treated with C118P for 72 h. The IC_50_ of C118P is summarised (**a**). The cell index was detected after MDA-MB-231 and MDA-MB-468 cells were treated with C118P at three concentrations (0.025, 0.05, 0.1 μM) or 0.5 μM taxol for 144 h by RTCA (**b**). Colony formation was detected for 2 weeks after MDA-MB-231 and MDA-MB-468 cells were treated with C118P (**c**). n = 3. The data are presented as the means ± S.D. of triplicate measurements and were analysed using Student’s *t*-test. *** *p* < 0.001, and ns represents no significant change vs. control group. The activation of proliferation-related proteins was tested by detecting mTOR, p-mTOR, p70S6K, and p-p70S6K in MDA-MB-231 and MDA-MB-468 (**d**) cells at 48 h, with β-actin serving as a loading control. The uncropped bolts are shown in Supplementary Materials.

We compared the viability of several breast cancer cell lines upon C118P treatment. Among these cell lines (MDA-MB-231, MDA-MB-468, BT-549, MCF-7, T47D, and BT-474 cells), C118P inhibited cell proliferation (Figure 1a), with IC_50_ values ranging from 9.35 to 325 nM. Then, RTCA showed that 0.025, 0.05, and 0.1 μM C118P treatment potently inhibited breast cancer cell proliferation for more than 3 days after C118P administration (Figure 1b). In addition, C118P significantly inhibited colony formation in the MDA-MB-231 and MDA-MB-468 cell lines (Figure 1c), with an inhibitory effect of more than 50% at 100 nM C118P. As reported previously [23], mTORC1 is a central regulator. It regulates cell growth, mRNA translation, and metabolism. Our data suggest that C118P treatment markedly decreased phosphorylated p70S6K and phosphorylated S6 levels (Figure 1d). Thus, we demonstrated that C118P inhibits breast cancer progression in vitro.

### 3.2. C118P Substantially Potentiated Apoptosis and Cell Cycle Arrest in Breast Cancer Cells

**Figure 2:**
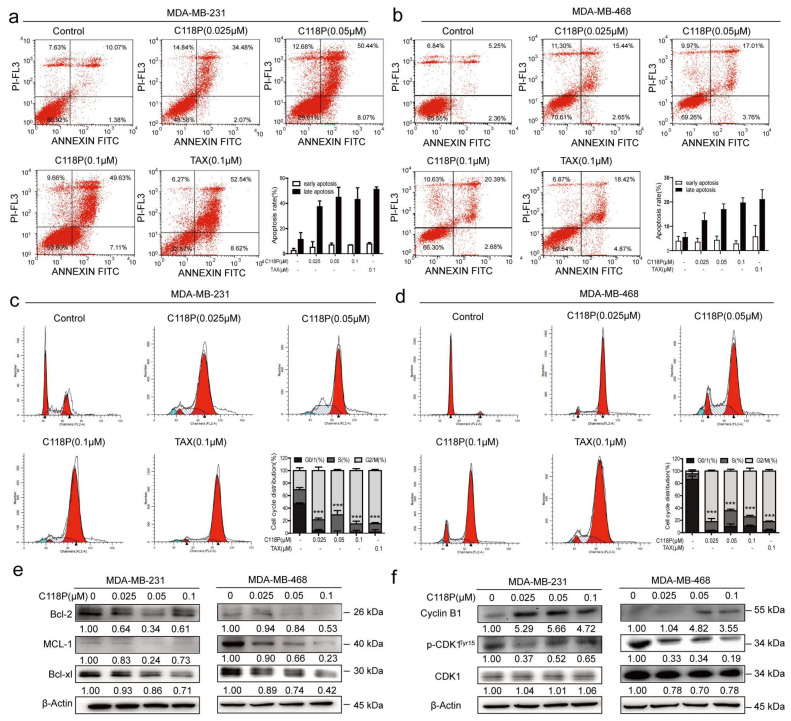
Treatment with C118P induced apoptosis and G2/M cell cycle arrest in breast cancer cells. MDA-MB-231 and MDA-MB-468 cells were treated with 0.025, 0.05, or 0.1 μM C118P or 0.1 μM taxol for 48 h. Annexin V/PI analysis of MDA-MB-231 (**a**) and MDA-MB-468 (**b**) cells was performed by flow cytometry to detect the percentage of apoptotic cells, and the frequency of apoptotic cells (including early and late apoptotic cells) is shown in the histograms. Activation of the apoptosis-related proteins Bcl-2, MCL-1, and Bcl-xl in MDA-MB-231 and MDA-MB-468 (**e**) cells was detected at 48 h, with β-actin serving as a loading control. The data are presented as the means ± S.D. of triplicate measurements. The percentages of MDA-MB-231 (**c**) and MDA-MB-468 (**d**) cells in each phase are shown in the histograms, and the data are presented as the means ± S.D. of triplicate measurements and were analysed using Student’s *t*-test to evaluate the statistical significance of differences in G2/M arrest. *** *p* < 0.001 vs. control group. n = 3. (**f**) Expression of the G2/M arrest-related proteins Cyclin B1, CDK1, and phosphorylated CDK1 (Tyr15) was analysed, with β-actin serving as a loading control. The uncropped bolts are shown in Supplementary Materials.

We performed Annexin-V/PI staining to detect apoptosis. The results indicated that the apoptosis rate increased in both the MDA-MB-231 and MDA-MB-468 cell lines following treatment with C118P (0.025 μM, 0.05 μM, or 0.1 μM) for 48 h compared to the corresponding controls (Figure 2a,b, *p* < 0.01). Bcl-2, Bcl-xl, and MCL-1 [24] have been identified as specific apoptosis markers [25]. Apoptosis induction by C118P was further shown by the decreased expression of Bcl-xl and MCL-1 upon C118P treatment (Figure 2e). In addition to apoptosis, cell cycle arrest was detected. As shown in Figure 2c,d, C118P induced cell cycle arrest at the G2/M phase in the MDA-MB-231 and MDA-MB-468 cell lines. Furthermore, the transition from the G2 phase requires activation of the Cyclin B1/CDK1 checkpoint complex. Here, we found that Cyclin B1 accumulated and that p-CDK1 was dramatically reduced after treatment with C118P (Figure 2f). Collectively, these results suggest that C118P inhibits proliferation by inducing cell cycle arrest at the G2/M phase and apoptosis in breast cancer cells.

### 3.3. Validation of ASCT2 As a Target of C118P through Reverse Docking, SPR, and MST Analyses

**Figure 3:**
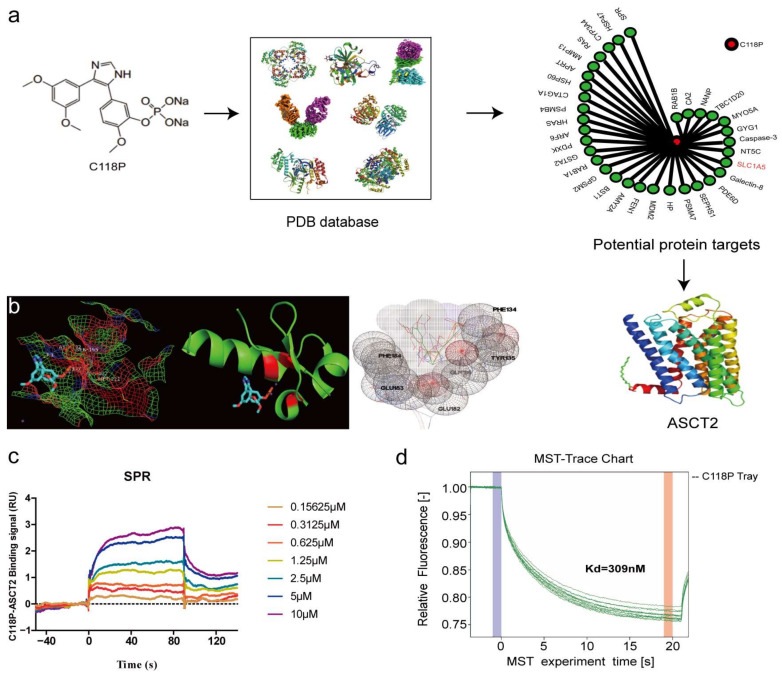
The structure of C118P and its binding affinity with ASCT2. (**a**) Chemical structure of C118P. By docking C118P with 100,000 protein crystal structures in the PDB database and comprehensive evaluation of drug target docking scores, ASCT2 was selected as a potential target for C118P. Then, ASCT2 (PDB: 5llm) was docked with C118P, the conformation with the lowest binding energy (−7.6 kcal/mol) was selected (**b**), and all amino acids within 1 Å of C118P were displayed with PyMOL software. (**c**,**d**) We further verified the binding affinity between C118P and ASCT2 by performing SPR and MST to detect the KD.

To identify the target of C118P, proteins in the PDB database were docked with C118P. Then, ASCT2 was selected from a reverse docking library as a potential target of C118P (Figure 3a). Additionally, AutoDock Vina software (version 1.0.8) was used to dock ASCT2 (PDB: 5llm) with C118P, and the conformation with the lowest binding energy (−7.6 kcal/mol) was then selected, and all of the amino acids less than 1 Å from C118P were shown with PyMOL software (version 1.5.3). As shown in Figure 3b, the data indicated that ASN182, SER195, MET221, and ASN222 comprise the C118P-binding pocket. Among these residues, ASN182 and ASN222 form hydrogen bonds with C118P. As ASCT2 is a transmembrane protein, obtaining full-length ASCT2 was very difficult. Therefore, we obtained an active fragment of ASCT2, as described in the Methods section. ASCT2 was then expressed, purified, and identified using Western blot analysis and Coomassie staining (Figure S1a,e). We next applied SPR analysis to investigate whether and how C118P directly interacts with ASCT2. The biochemical parameters for the binding of C118P and ASCT2 were then measured. Our results showed that the equilibrium dissociation constant (KD) of C118P toward ASCT2 is 2.358 μM (Figure 3c). The MST analysis showed that the KD of C118P toward ASCT2 is 309 nM (Figure 3d and Figure S1f). This finding indicated that ASCT2 is a potential target of C118P.

### 3.4. C118P Inhibits Glutamine Metabolism and Mediates Autophagy in Breast Cancer Cells

**Figure 4:**
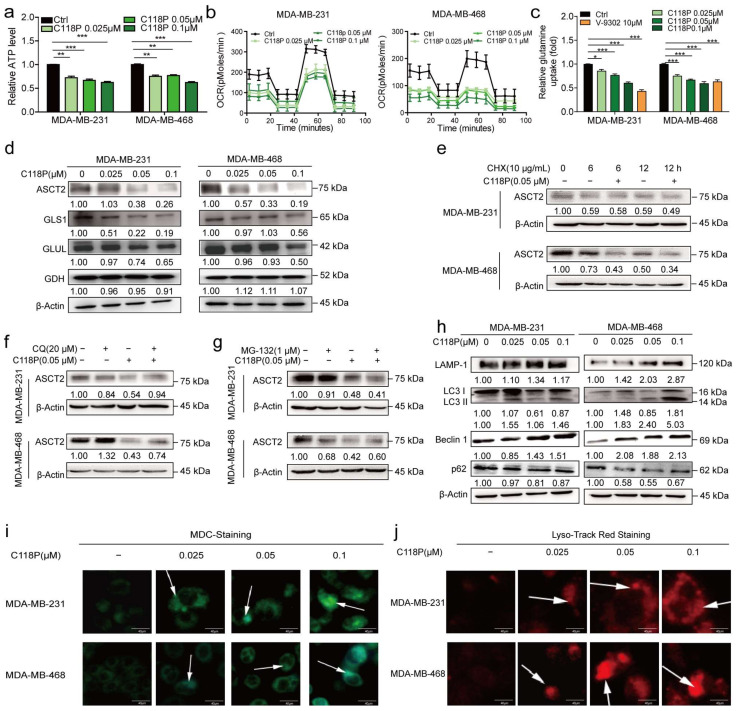
C118P inhibited glutamine metabolism and induced the degradation of ASCT2. MDA-MB-231 and MDA-MB-468 cells were treated with 0.025, 0.05, or 0.1 μM C118P and 10 μM V-9302 for 48 h. Relative ATP production (**a**), the OCR (**b**), and relative glutamine uptake (**c**) were detected, and the data are presented as the means ± S.D. of triplicate measurements and were analysed using Student’s *t*-test. * *p* < 0.05, ** *p* < 0.01, and *** *p* < 0.001 vs. control group. n = 3. (**d**) ASCT2, GLS1, GLUL, and GDH protein levels in MDA-MB-231 and MDA-MB-468 cells were determined using Western blotting, and β-actin served as a loading control. (**e**) Cells were treated with 10 μg/mL CHX and vehicle or 0.05 μM C118P for 6/12 h. Then, cell lysates were collected for Western blot analysis of ASCT2. Cells were coincubated with the proteasome inhibitor CQ (20 μM) (**f**) or MG-132 (1 μM) (**g**). After pretreatment with C118P (0.05 μM) for 48 h, the expression of ASCT2 was detected by Western blotting. β-Actin served as a loading control. (**h**) Autophagy-related markers (LAMP-1, LC3 I/II, Beclin1, and p62) were detected after MDA-MB-231 and MDA-MB-468 cells were treated with 0.025, 0.05, or 0.1 μM C118P for 48 h. (**i**) Autophagosomes in MDA-MB-231 and MDA-MB-468 cells were labelled with MDC. (**j**) Lysosomes were labelled with LysoTracker Red fluorescence dye. Scale bars, 40 μm. The uncropped bolts are shown in Supplementary Materials.

ASCT2, the primary glutamine transporter, is overexpressed in different cancers [5]. An analysis of the TCGA database revealed that ASCT2 is expressed at high levels in basal-like breast cancer, and distinct associations exist between ASCT2 expression and the prognosis of patients with breast cancer (Figure S2a–d). Thus, we hypothesised that C118P inhibits glutamine metabolism by targeting ASCT2. As expected, at concentrations of 0.025 μM, 0.05 μM, and 0.1 μM, C118P reduced ATP production (Figure 4a). We also investigated the dependency of MDA-MB-231/MDA-MB-468 cells on oxidative phosphorylation (OXPHOS) by measuring the oxygen consumption rate (OCR) with a real-time metabolite analyser. The basal OCR was much lower after treatment with C118P, implying that C118P inhibits the metabolism of breast cancer cells (Figure 4b). Then, a glutamine uptake assay showed that C118P inhibits the uptake of glutamine (Figure 4c). In addition to ASCT2, several key metabolic enzymes, such as GLS1, GLUL, and GDH, are involved in glutamine metabolism. Interestingly, we found that ASCT2 protein expression, but not mRNA expression, was reduced (Figure 4d and Figure S3a). As shown in Figure 4e, after treatment with cycloheximide (CHX, 10 μg/mL), ASCT2 was still degraded. This result indicates that C118P mediates the degradation of ASCT2. Proteolysis in eukaryotic cells is mainly mediated by the ubiquitin (Ub)-proteasome system (UPS) [26] and autophagosome system [27]. As shown in Figure 4f,g, only the lysosome inhibitor chloroquine (CQ), but not the proteasome inhibitor MG-132, reversed the reduction in ASCT2 levels caused by C118P. This means that ASCT2 is degraded through the lysosomal pathway. There are three types of biomarkers in the process of autophagy: markers of vesicle formation, lysosomal markers, and markers of autophagic substrates. During vesicle formation, LC3, which exists in the forms LC3-I and LC3-II as a result of LC3-I transformation, is involved in the formation of autophagosome membranes [28]. Lysosome-associated membrane protein type 1 (LAMP-1) is a lysosomal marker [29]. As shown in our study, C118P induced autophagy in breast cancer cells and increased expression of the autophagy markers LAMP-1, LC3-II, and Beclin1. Meanwhile, the expression of p62 was decreased (Figure 4h and Figure S3b–f). Previous reports have suggested that MDC accumulates in mature autophagic vacuoles, such as autophagolysosomes, and autophagic vacuoles stained with MDC appear as distinct dot-like structures distributed in the cytoplasm [30]. Data obtained from MDC staining and LysoTracker Red staining showed that autophagosomes emerged after C118P treatment (Figure 4i,j and Figure S3g,h).

### 3.5. C118P Inhibits Breast Cancer Metabolism via ASCT2

**Figure 5:**
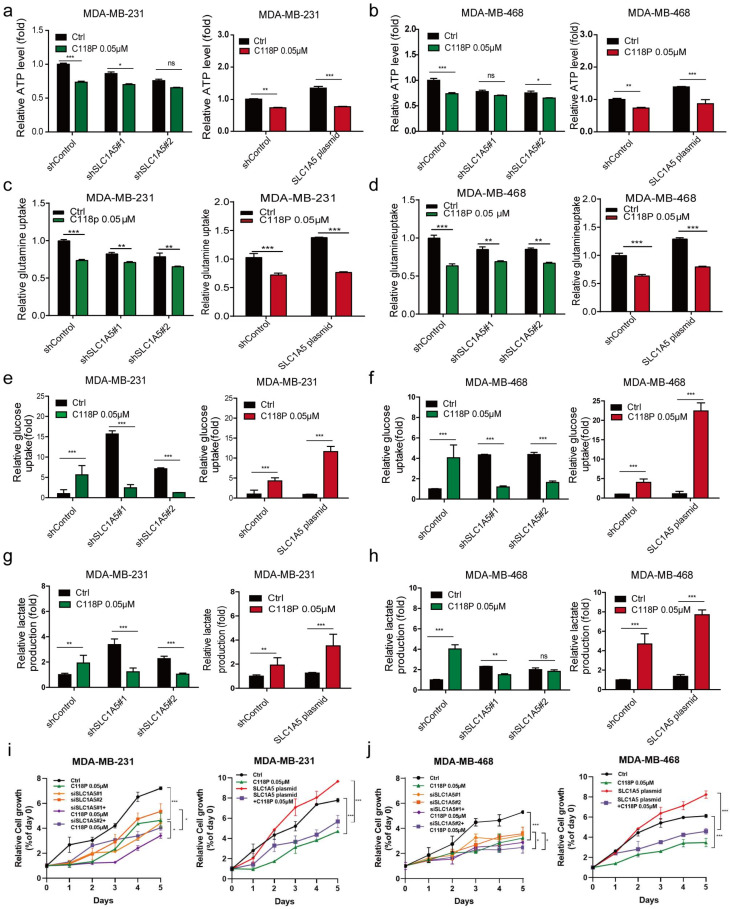
C118P inhibited glutamine metabolism via ASCT2 in vitro. In ASCT2-knockdown and ASCT2-overexpressing cell lines, the effects of C118P on ATP production (**a**,**b**), glutamine uptake (**c**,**d**), glucose uptake (**e**,**f**), and lactate production (**g**,**h**) were detected. The cell growth curve was detected after MDA-MB-231 and MDA-MB-468 cells were treated with C118P at 0.05μM with or without si*SLC1A5*#1, si*SLC1A5*#2, and *SLC1A5* plasmid for 120 h (**i**,**j**). n = 3. The data are presented as the means ± S.D. of triplicate measurements and were analysed using Student’s *t*-test. * *p* < 0.05, ** *p* < 0.01, *** *p* < 0.001, and ns represents no significant change vs. shControl group.

Since we found that C118P reduces the production of ATP and uptake of glutamine, we investigated whether C118P inhibits breast cancer metabolism via ASCT2. First, we constructed stable ASCT2-knockdown and ASCT2-overexpressing cell lines (Figure S4a–d). Then, the different metabolic responses of these cells after pretreatment with C118P were investigated. The data showed that after treatment with C118P, the metabolic response of cells that overexpressed ASCT2 was significantly different than that of cells in which ASCT2 had been knocked down. After treatment with C118P, the inhibition of ATP production ranged from 50% to 60% in ASCT2-knockdown cells, compared to 135% to 145% in ASCT2-overexpressing cells (Figure 5a,b). The inhibition of glutamine uptake ranged from 46% to 63% in ASCT2-knockdown cells, compared to 130% to 145% in ASCT2-overexpressing cells (Figure 5c,d). The promotion of the glucose uptake and lactate production by C118P was reversed in the ASCT2-knockdown group (Figure 5e–h). The growth curve results showed that although overexpressed ASCT2 slightly rescued the survival of breast cancer cells after C118P treatment, relatively speaking, we found that compared with cells in the negative control group, at the same concentration of C118P, the inhibitory effect on cells overexpressing ASCT2 was still strong, while the inhibitory effect on cells with the knockout of ASCT2 was the weakest (Figure 5i,j). 

In general, these results indicate that the *SLC1A5*-overexpressing group was more susceptible to C118P pretreatment than the shControl-transfected group. However, the *SLC1A5*-knockdown group was insensitive to C118P. In conclusion, C118P inhibits breast cancer metabolism via ASCT2.

### 3.6. Adipocytes Upregulated ASCT2 Expression in Breast Cancer Cells through IL-6

**Figure 6:**
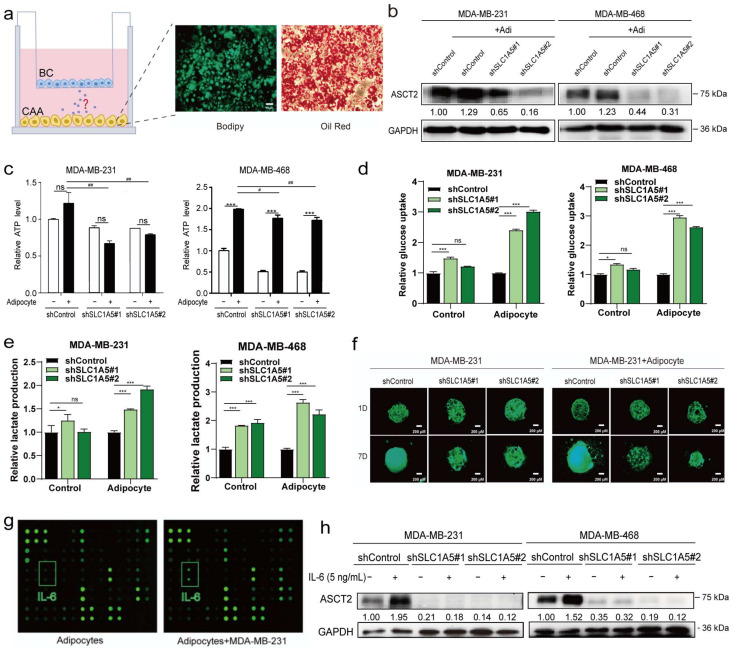
Adipocytes upregulated ASCT2 expression in breast cancer cells through IL-6. Bodipy staining and oil red O staining were performed to assess the differentiation efficiency of adipocytes (**a**). Scale bars, 50 μm. After co-culture with adipocytes, the expression of ASCT2 was detected using Western blotting. GAPDH served as a loading control (**b**). The effects of C118P on ATP production (**c**), glucose uptake (**d**), and lactate production (**e**) were detected in ASCT2-knockdown cell lines co-cultured with or without adipocytes. The data are presented as the means ± S.D. of triplicate measurements and were analysed using Student’s *t*-test. * *p* < 0.05, *** *p* < 0.001, and ns represents no significant change vs. control group. # *p* < 0.05 and ## *p* < 0.01 vs. adipocyte group. n = 3. ASCT2-knockdown cells were cultured in a 3D system, and photos were captured for 7 consecutive days (**f**). Scale bars, 200 μm. Cytokine contents in adipocytes and co-culture models were determined using a mouse microarray (**g**). After stimulation with IL-6 (5 ng/mL), ASCT2 expression was detected in ASCT2-knockdown cell lines using Western blotting. GAPDH served as a loading control (**h**). The uncropped bolts are shown in Supplementary Materials.

We constructed a co-culture model to investigate the interaction between breast cancer cells and adipocytes. A well-known “cocktail therapy” was used to induce the differentiation of 3T3-L1 cells into adipocytes. After incubating confluent cells with the differentiation medium for up to 14 days, a large number of lipid droplets formed and were observed under a bright-field microscope. Then, Bodipy staining and oil red O staining were used to confirm the differentiation efficiency, as shown in Figure 6a. These differentiated mature adipocytes were seeded in the lower chamber, and the triple-negative breast cancer cell lines MDA-MB-231 and MDA-MB-468 were seeded into the upper chamber. Then, a co-culture model system to mimic the adipocyte-rich breast cancer microenvironment was constructed. Co-culture with adipocytes upregulated ASCT2 expression, increased ATP levels, and promoted breast cancer cell proliferation (Figure 6b–f and Figure S5a). ASCT2 knockdown reversed these changes. These data verified that adipocytes promote glutamine metabolism via the glutamine transporter ASCT2 and suggested that ASCT2 might play an important role in the development of breast cancer. The microarray analysis showed that the levels of many cytokines in the supernatant changed significantly after co-culture; IL-6 was the main secreted protein and the cytokine detected at the highest levels (Figure 6g). The expression of the ASCT2 protein was significantly upregulated after IL-6 stimulation, while when ASCT2 was knocked down, the effect of IL-6 was reversed (Figure 6h). Based on these results, IL-6, secreted by the adipocytes, promotes breast cancer cell growth and metabolism by upregulating ASCT2 protein expression.

### 3.7. C118P Exerted Antitumour Effects on the Co-Culture of Breast Cancer Cells and Adipocytes via ASCT2

**Figure 7:**
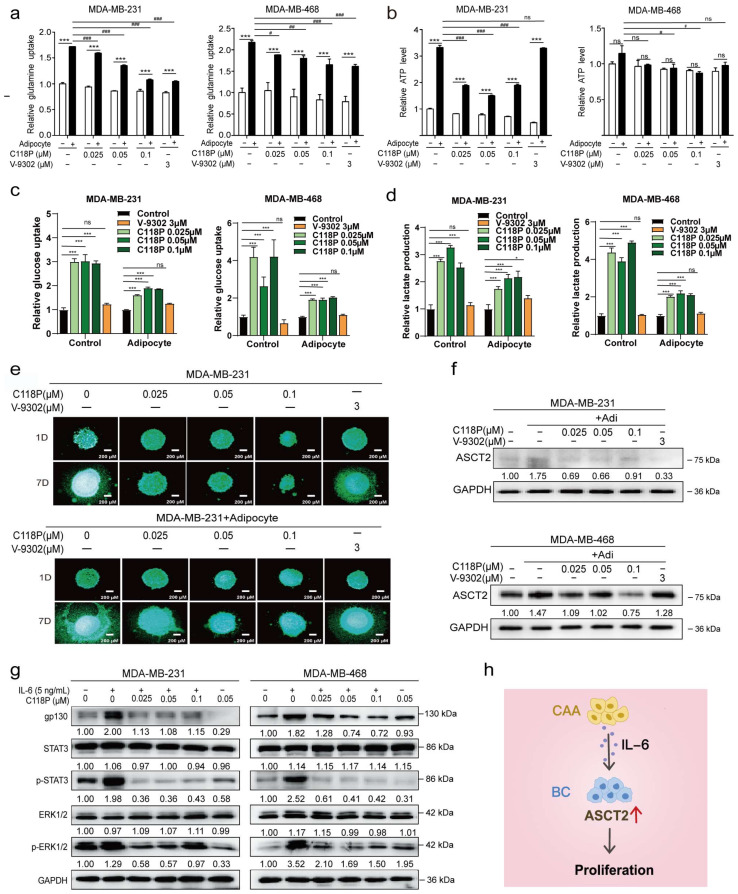
C118P exerted antitumour effects on the co-culture system of breast cancer cells and adipocytes through ASCT2. Breast cancer cells were treated with 0.025, 0.05, or 0.1 μM C118P or 3 μM V-9302 for 48 h. Relative glutamine uptake (**a**), relative ATP production (**b**), relative glucose uptake (**c**), and the lactate production (**d**) were detected in cells co-cultured with or without adipocytes, and the data are presented as the means ± S.D. of triplicate measurements and were analysed using Student’s *t*-test. * *p* < 0.05 and *** *p* < 0.001 vs. control group. # *p* < 0.05, ## *p* < 0.01, ### *p* < 0.001 vs. adipocyte group, and ns represents no significant change. n = 3. Breast cancer cells were treated with 0.025, 0.05, or 0.1 μM C118P or 3 μM V-9302 cultured in a 3D model, and photos were captured for 7 consecutive days (**e**). In the co-culture system, ASCT2 expression was detected using Western blotting after treatment with 0.025, 0.05, or 0.1 μM C118P or 3 μM V-9302 for 48 h. GAPDH served as a loading control (**f**). After stimulation with IL-6 (5 ng/mL) and 0.025, 0.05, or 0.1 μM C118P, the levels of gp130, STAT3, p-STAT3, ERK1/2, and p-ERK1/2 were detected in breast cancer cells using Western blotting. GAPDH served as a loading control (**g**). Adipocytes upregulate ASCT2 expression in breast cancer cells by secreted IL-6, which subsequently promotes tumour growth. The red arrow indicates upregulation (**h**). The uncropped bolts are shown in Supplementary Materials.

We investigated whether C118P inhibited breast cancer metabolism via ASCT2 in the co-culture system. C118P inhibited glutamine uptake and ATP generation, inhibited breast cancer cell proliferation, and downregulated ASCT2 expression in the co-culture system (Figure 7a–f and Figure S5b). The *SLC1A5* knockdown group was insensitive to C118P. The effect of C118P in the co-culture system on ATP production, glucose uptake, and lactate production in knockdown cells was weaker than that in the control group (Figure S6a–c). The expression of IL-6 receptor gp130 was upregulated after IL-6 stimulation. Meanwhile, the levels of p-STAT3 and p-ERK1/2 were increased. However, C118P treatment combined with IL-6 stimulation inhibited the upregulation of gp130 (Figure 7g,h).

### 3.8. C118P Exerted Antitumour Effects via ASCT2 In Vivo

**Figure 8:**
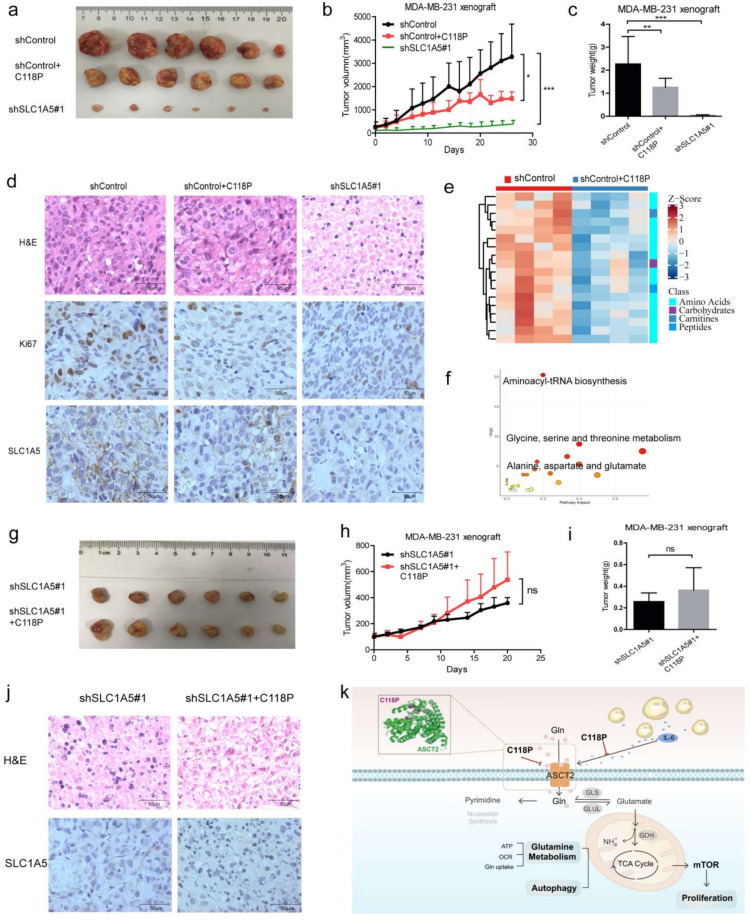
C118P suppressed tumour growth via ASCT2 in MDA-MB-231 xenograft nude mice. In the MDA-MB-231 xenograft nude mouse model, when tumours grew to 100–200 mm^3^, mice were administered 50 mg/kg C118P (i.v., q.o.d., 4 weeks). Images of the tumours (**a**) are shown. Statistical analyses of the tumour volume (**b**) and tumour weight (**c**) were performed. MDA-MB-231 cells transfected with sh*SLC1A5* were used as a positive control. The data are expressed as the mean ± S.D. and were analysed using Student’s *t*-test. * *p* < 0.05, ** *p* < 0.01, and *** *p* < 0.001. H&E staining and immunohistochemical staining for *SLC1A5* and Ki67 (**d**). Targeted metabolomics analysis. Enrichment analysis of the differentially altered metabolites and pathways (**e**,**f**). In the MDA-MB-231 sh*SLC1A5* xenograft nude mouse model, when tumours grew to 100–200 mm^3^, mice were administered 50 mg/kg C118P (i.v., q.o.d., 3 weeks). Magnified views of the tumour (**g**) are shown. Statistical analyses of tumour volume (**h**) and tumour weight (**i**) were performed. H&E staining and immunohistochemical staining for *SLC1A5* are shown (**j**). Scale bars, 50 μm. (**k**) The mechanism by which C118P suppresses breast cancer proliferation via ASCT2. Schematic representation of the relationship between C118P and ASCT2.

Previous data indicated that C118P represses the proliferation of breast cancer in vitro; thus, we further investigated whether C118P exerts anti-breast cancer effects via ASCT2. In MDA-MB-231 nude mouse xenografts, C118P (50 mg/kg, i.v.) significantly inhibited tumour growth (*p* < 0.01) (Figure 8a–c). As shown in Figure 3, ASCT2 was confirmed as a target of C118P, so we aimed to detect the effect of C118P when ASCT2 was knocked down. Interestingly, when ASCT2 was knocked down, C118P (50 mg/kg, i.v.) did not exert a clear anti-breast cancer effect on MDA-MB-231 xenografts in nude mice (Figure 8g–i). Additionally, H&E staining and immunohistochemical staining detected *SLC1A5* and Ki67 (Figure 8d,j and Figure S7a–c). The metabolomic analysis showed that C118P significantly inhibited amino acid metabolism and lipid metabolism in tumour tissues (Figure 8e,f). These results indicate that ASCT2 is a target of C118P (Figure 8k).

Taken together, the study findings show that C118P significantly exerted antitumour effects via ASCT2 in vitro and in vivo, providing evidence to confirm that ASCT2 might be an effective target in the clinical therapy of breast cancer.

## 4. Discussion

Targeting metabolic abnormalities is a research direction that has attracted much attention in the field of novel antitumour drug development. Recently, glutamine metabolism has become a hotspot in tumour metabolism research. As a transporter for glutamine, ASCT2 is an attractive tumour metabolism target, based on its role and increased physical activity in cancer [31]. The inhibitors targeting ASCT2 mainly consist of small molecules and antibodies, such as MEDI7247, GPNA, V9302, etc. Despite these ASCT2 inhibitors, so far, few patients have benefited from ASCT2 inhibitor treatment strategies. In our study, C118P bound to ASCT2 and decreased its expression. Therefore, ASCT2 is recognised as a target of C118P. Compared with other ASCT2 inhibitors, C118P has the advantages of high efficiency and low toxicity.

The ASCT2 inhibitor MEDI7247 is a novel pyrrolobenzodiazepine antibody-drug conjugate (ADC) monoclonal antibody. MEDI7247 showed potent activity in vitro and in vivo in a spectrum of haematological cancers and solid tumours. GPNA is widely used as a drug to inhibit ASCT2 in basic research (IC_50_~1000 μM) [13]. However, as an ASCT2 inhibitor, GPNA exhibits poor potency and selectivity in human cells. V-9302 was reported to be the first specific and potent small-molecule inhibitor of ASCT2. V-9302 was shown to significantly inhibit ASCT2-mediated glutamine uptake with an IC_50_ of 9.6 μM [14]. Suppression of ASCT2 by V-9302 resulted in attenuated proliferation of cancer cells and increased oxidative stress, which, collectively, contributed to antitumour responses in vitro and in vivo. Nevertheless, V-9302 has shortcomings, such as low selectivity and high toxicity. Research showed [14] that the response to V-9302 did not correlate with the level of ASCT2 expression in tumours. This means that the results of studies in which patients with high expression of the target ASCT2 are enrolled will be unreliable. 

Here, we report the anti-breast cancer effects of C118P. C118P inhibited the proliferation of breast cancer cell lines with an IC_50_ of 9.35 to 325 nM and exhibited an improvement in potency over GPNA and V-9302. Moreover, C118P exposure resulted in decreased mTOR activity, which is consistent with reduced amino acid transport and metabolism. *SLC1A5* silencing was reported to inhibit oesophageal cancer growth by inducing cell cycle arrest and apoptosis [32]. C118P potentiated cell apoptosis and G2/M cell cycle arrest in breast cancer cells, and these effects might be mediated by ASCT2. 

In this study, we identified ASCT2 as a target of C118P. Because of the structural complexity of membrane proteins, Jeff Holst [33] constructed a homology model of ASCT2 for virtual screening [34]. Benefitting from the resolution of the crystal structure of ASCT2 [35], we obtained possible binding sites between the drug C118P and ASCT2 via virtual screening, which is worthy of further study in the future. Experimental validation of the specific binding sites of C118P with ASCT2, such as point mutation experiments and molecular dynamics simulations, may be necessary. C118P is currently being investigated in a phase I clinical trial in China. We hope our results guide the application of ASCT2 as a target of C118P in subsequent phase II clinical trials. 

We found that the metabolic regulatory effect of C118P may not be closely related to the glutamine metabolic enzymes. Meanwhile, C118P may affect protein stability rather than protein synthesis after binding to ASCT2. Further research found that ASCT2 may be degraded through the autophagy–lysosomal pathway. Elevated autophagy is another notable characteristic of the observed C118P-mediated response. However, more research on this topic needs to be undertaken before the mechanism of ASCT2 downregulation can be more clearly understood. C118P probably exerts a dual effect on ASCT2. C118P binds directly to ASCT2, thus inhibiting the transport of amino acids, such as glutamine, and promoting ASCT2 degradation. Moreover, in the tumour microenvironment, C118P may indirectly affect ASCT2 expression through IL-6 and receptor gp130. Further experiments are still needed to verify this conclusion. 

Despite these promising results, questions remain. Proteins were docked with C118P to identify the targets. Our results showed that the antitumour effect of C118P was partly caused by ASCT2-mediated metabolic alterations. However, other targets of C118P are not excluded. C118P showed antitumour effects against melanoma via BUB1B or against HCC via tumour vasculature [36,37]. In addition, our other studies have shown that C118P inhibited breast cancer metastasis through ASCT2. Whether C118P exerts antitumour effects through other targets in breast cancer still needs further investigation. Another significant issue is that specific antagonism of ASCT2 will also block the ASCT2-mediated transport of other neutral amino acids beyond glutamine. It cannot be excluded that the observed efficacy may be partly due to simultaneous blockade of multiple ASCT2 substrates. 

In terms of experimental design, we used a cell-derived xenograft (CDX) model to evaluate the in vivo efficacy of C118P. However, because the CDX model cannot maintain the heterogeneity of tumour tissue, its biological characteristics and drug efficacy evaluation results are less similar to clinical characteristics. The patient-derived xenograft (PDX) model retains tumour heterogeneity, is more consistent with clinical tumour characteristics, and has better clinical predictability. We will continue to further evaluate the in vivo efficacy of C118P in the PDX model to provide a basis for clinical research. Moreover, expanding the sample size in future studies will allow us to more fully evaluate the efficacy and safety of C118P.

Targeting ASCT2 provides a new option for tumour therapy, but due to the existence of tumour metabolic heterogeneity, not all patients can benefit from ASCT2 inhibition, interference with glutamine metabolism treatment strategies. Finding sensitive indications for the clinical mediation of ASCT2 inhibitors is a common problem faced by targeted metabolism antitumour drugs, which has become an urgent issue to be solved. In the future, in view of the above problems, the metabolic heterogeneous characteristics of breast cancer and glutamine dependence will be analysed to find the difference in drug sensitivity and causes of ASCT2 inhibitors.

## 5. Conclusions

In summary, we found that C118P regulates glutamine metabolism by inhibiting glutamine uptake through ASCT2, resulting in antitumor effects. Our conclusion proposed a prospect that ASCT2 might be a candidate target of C118P in breast cancer treatment. ASCT2 may be a promising therapeutic target for tumours, especially glutamine-sensitive tumours.

## Data Availability

All relevant data are within the paper and its Supplementary Materials. The data that support the findings of this study are available from the corresponding author upon reasonable request.

## Supplementary Materials

The following supporting information can be downloaded at: https://www.mdpi.com/article/10.3390/cancers15205082/s1. Supplementary material associated with this article can be found in the online version. Figure S1: ASCT2 was expressed in E. coli and purified. The ASCT2 protein was validated by western blotting and Coomassie staining. (a,b) SDS-PAGE and Western Blot analysis of the expression of ASCT2 proteins with the change of IPTG concentration. (c–e) Purification of ASCT2 through Ni+ column and AKTA pure. (f) C118P-ASCT2 interactions were measured by MST. Figure S2: Expression level of ASCT2 in breast cancer and survival analysis. (a,c) The expression of the SLC1A5 in different cancers or specific cancer subtypes was analysed using GEPIA2 and The Human Protein Atlas. (b) Based on TCGA data, the expression level of the SLC1A5 was analysed in the main pathological stages (stage I, stage II, stage III, stage IV, and stage V) of BRCA. Log2 (TPM + 1) was applied for log transformation. (d) We used GEPIA2 to analyse the OS of patients with breast cancer in TCGA stratified by SLC1A5 expression. Figure S3: C118P inhibited glutamine metabolism and induced autophagy in vitro. (a) ASCT2, GLS1, GLUL, and GDH mRNA levels were measured by qRT-PCR in MDA-MB-231 and MDA-MB-468 cells treated with C118P 0.05 μM for 48 h. (b–f) LAMP-1, MAP1LC3A, MAP1LC3B, Beclin1, and p62 mRNA levels were measured by qRT-PCR in MDA-MB-231 and MDA-MB-468 cells treated with C118P 0.025 μM, 0.05 μM, and 0.1 μM for 48 h, respectively. (g,h) The aotophagosome density and the lysosome density was analysed in MDA-MB-231 and MDA-MB-468 cells treated with C118P 0.025 μM, 0.05 μM, and 0.1 μM for 48 h, respectively. n = 3. The data are presented as the means ± S.D. of triplicate measurements and were analysed using Student’s *t*-test. * *p* < 0.05, ** *p* < 0.01, *** *p* < 0.001, and ns represents no significant change vs. control group. Figure S4: The transfection efficiency for ASCT2 knockdown and overexpression. Western blot analysis was performed to verify the transfection efficiency for ASCT2 knockdown (a) and overexpression (b) in the MDA-MB-231 and MDA-MB-468 cell lines. The lower panel shows the statistical data. The ASCT2 mRNA level upon ASCT2 knockdown (c) and overexpression (d) was determined in the MDA-MB-231 and MDA-MB-468 cell lines. n = 3. The data are presented as the means ± S.D. of triplicate measurements and were analysed using Student’s *t*-test. *** *p* < 0.001 vs. shControl group. Figure S5: C118P inhibited breast cancer cell proliferation in the co-culture system. (a) ASCT2-knockdown cells were cultured in a 3D system and photos were captured for 7 consecutive days. (b) Breast cancer cells were treated with 0.025, 0.05, or 0.1 μM C118P, cultured in a 3D model, and photos were captured for 7 consecutive days. Figure S6: C118P inhibiting cell metabolism of human breast cancer cells in cocluture system. Human breast cancer cells and ASCT2-knockdown cells were co-cultured for three days in the presence or absence of adipocytes. Different doses of C118P were added in the culture medium for 48 h. ATP production (a), glucose uptake (b) and lactate production (c) were detected after treatment with C118P for 48 h in human breast cancer cells. Quantitative Results are representative of three independent experiments mean ± SD. Data are analyzed using the Student’s *t*-test was statistically significant; ** *p* < 0.01, *** *p* < 0.001 vs. shControl group. Figure S7: The statistical analysis of Ki67 positive cells and ASCT2 positive cells related to Figure 8. (a–c) The statistical analysis of Ki67, ASCT2 and Ki67, respectively. Data are analyzed using the Student’s *t*-test was statistically significant; *** *p* < 0.001 vs. shControl group.
