# Supplementary material for: A Novel ASCT2 Inhibitor, C118P, Blocks Glutamine Transport and Exhibits Antitumour Efficacy in Breast Cancer
Source: Cancers (Basel). 2023 Oct 20;15(20):5082. doi: 10.3390/cancers15205082 (PMC10605716; doi:10.3390/cancers15205082)
Supplement: Supplementary file 1 [file cancers-15-05082-s001.zip › cancers-2622480-supplementary.pdf]

Supplementary Data (figures)

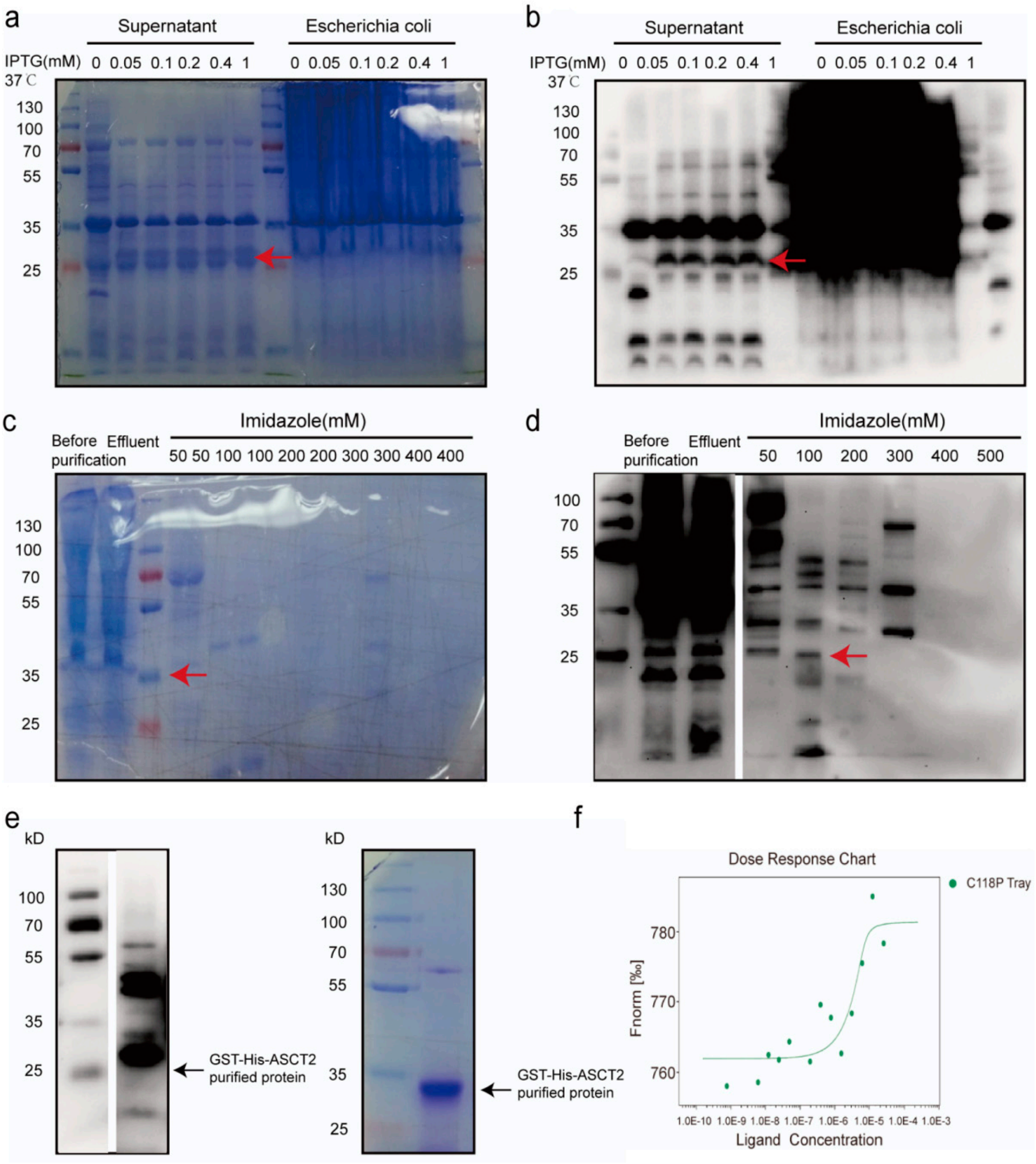

Figure S1: ASCT2 was expressed in *E. coli* and purified. The ASCT2 protein was validated by western blotting and Coomassie staining. (a-b) SDS-PAGE and Western Blot analysis of the expression of ASCT2 proteins with the change of IPTG concentration. (c-e) Purification of ASCT2 through Ni+ column and AKTA pure. (f) C118P-ASCT2 interactions were measured by MST.

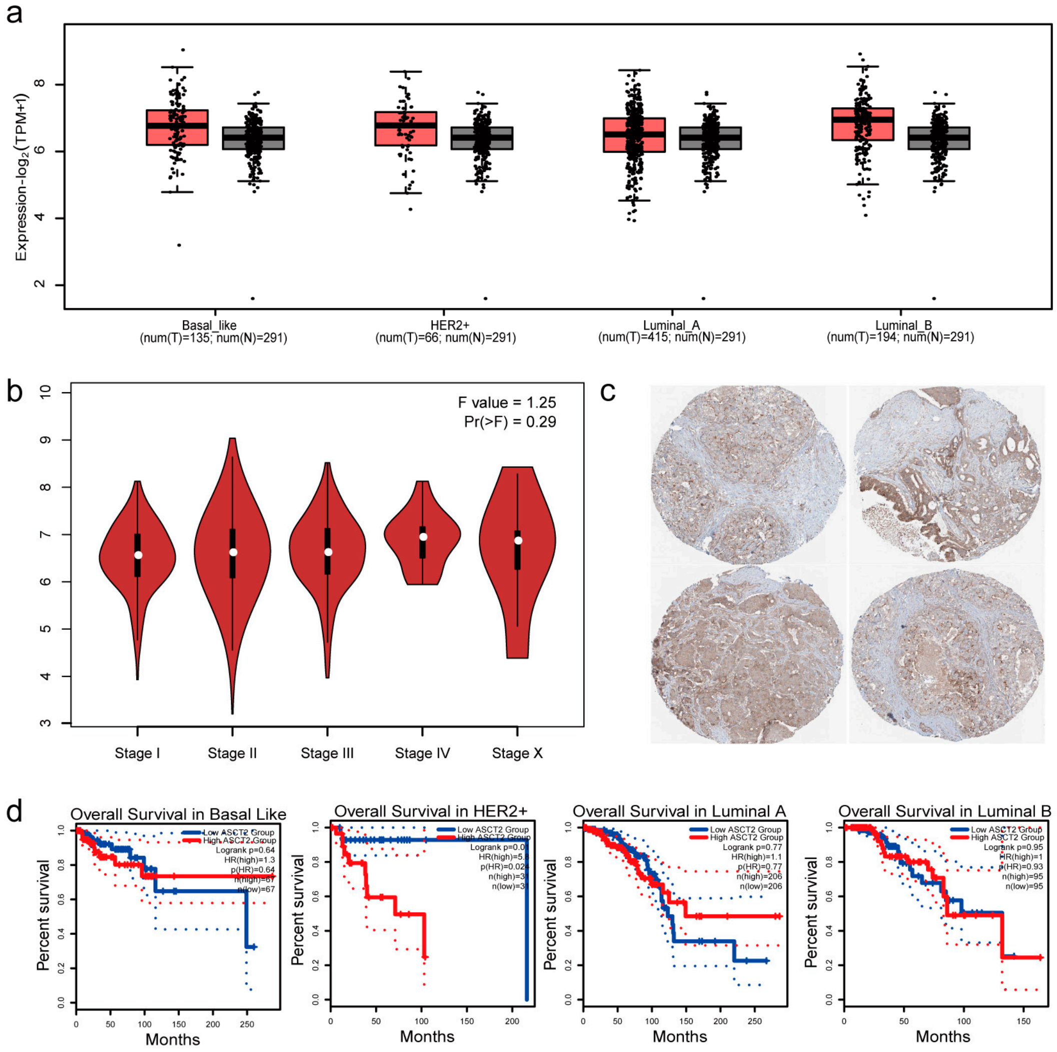

Figure S2: Expression level of ASCT2 in breast cancer and survival analysis. (a, c) The expression of the *SLC1A5* in different cancers or specific cancer subtypes was analysed using GEPIA2 and The Human Protein Atlas. (b) Based on TCGA data, the expression level of the *SLC1A5* was analysed in the main pathological stages (stage I, stage II, stage III, stage IV, and stage V) of BRCA. Log2 (TPM + 1) was applied for log transformation. (d) We used GEPIA2 to analyse the OS of patients with breast cancer in TCGA stratified by *SLC1A5* expression.

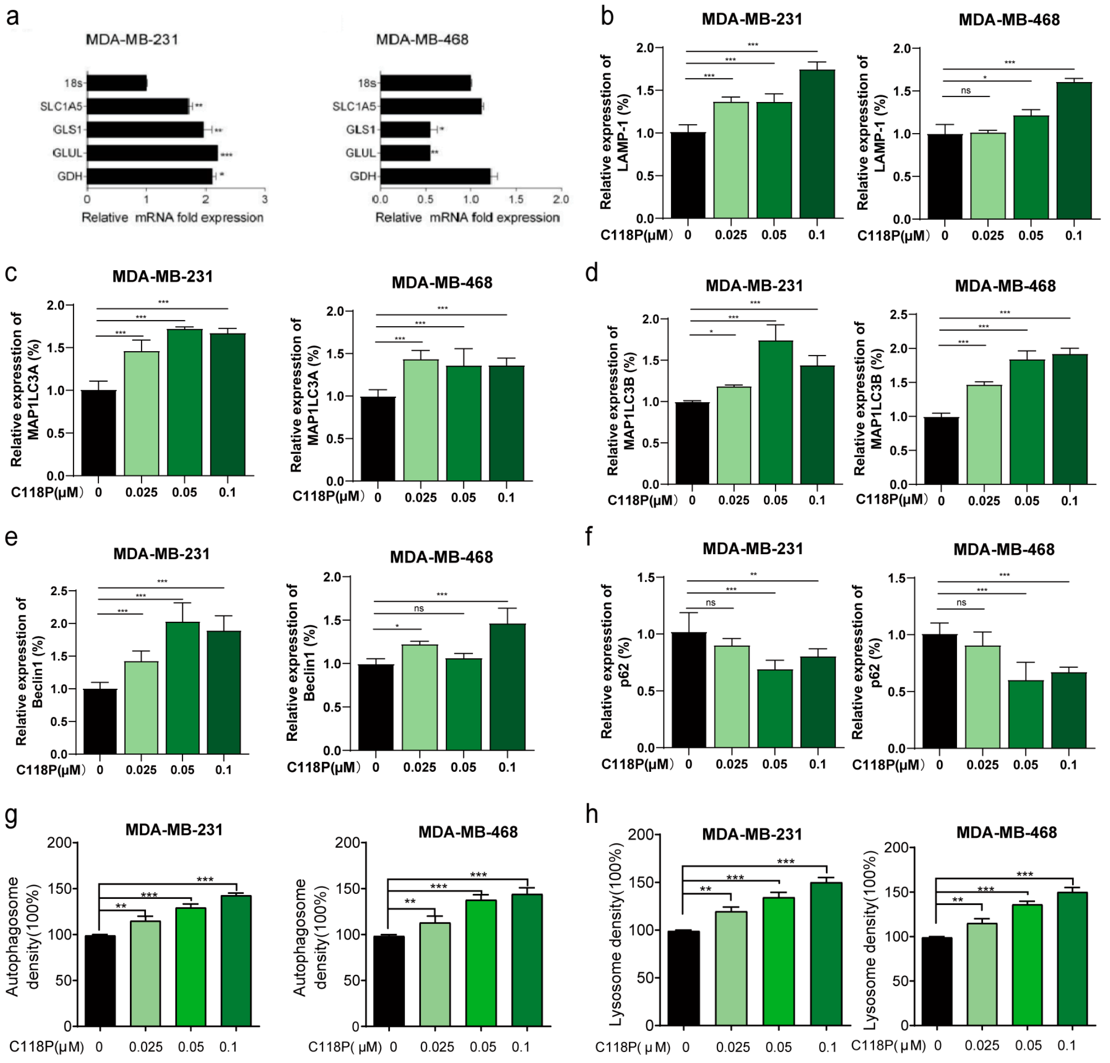

Figure S3: C118P inhibited glutamine metabolism and induced autophagy in vitro. (a) ASCT2, GLS1, GLUL, and GDH mRNA levels were measured by qRT-PCR in MDA-MB-231 and MDA-MB-468 cells treated with C118P 0.05 μM for 48 h. (b-f) LAMP-1, MAP1LC3A, MAP1LC3B, Beclin1, and p62 mRNA levels were measured by qRT-PCR in MDA-MB-231 and MDA-MB-468 cells treated with C118P 0.025 μM, 0.05 μM, and 0.1 μM for 48 h, respectively. (g-h) The aotophagosome density and the lysosome density was analysed in MDA-MB-231 and MDA-MB-468 cells treated with C118P 0.025 μM, 0.05 μM, and 0.1 μM for 48 h, respectively. n=3. The data are presented as the means ± S.D. of triplicate measurements and were analysed using Student's t-test. *P < 0.05, **P < 0.01, ***P < 0.001, and ns represents no significant

change vs. control group.

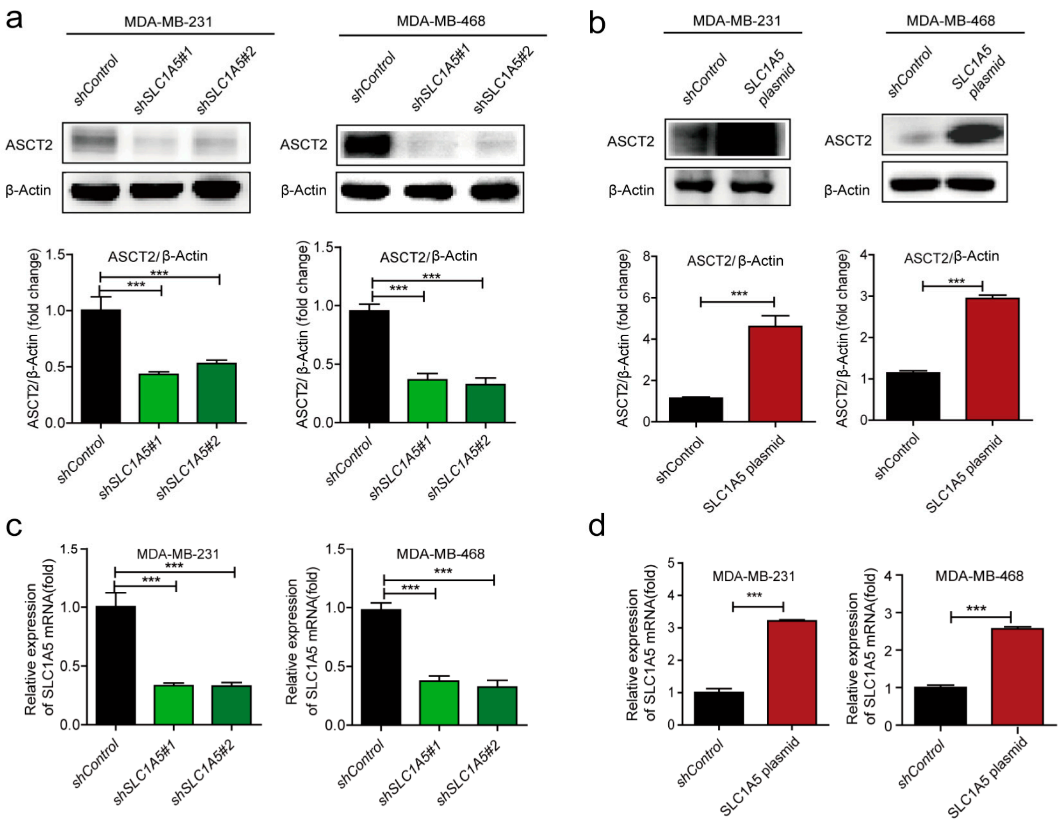

Figure S4: The transfection efficiency for ASCT2 knockdown and overexpression. Western blot analysis was performed to verify the transfection efficiency for ASCT2 knockdown (a) and overexpression (b) in the MDA-MB-231 and MDA-MB-468 cell lines. The lower panel shows the statistical data. The ASCT2 mRNA level upon ASCT2 knockdown (c) and overexpression (d) was determined in the MDA-MB-231 and MDA-MB-468 cell lines. n=3. The data are presented as the means ± S.D. of triplicate measurements and were analysed using Student's t-test. ***P < 0.001 vs. shControl group.

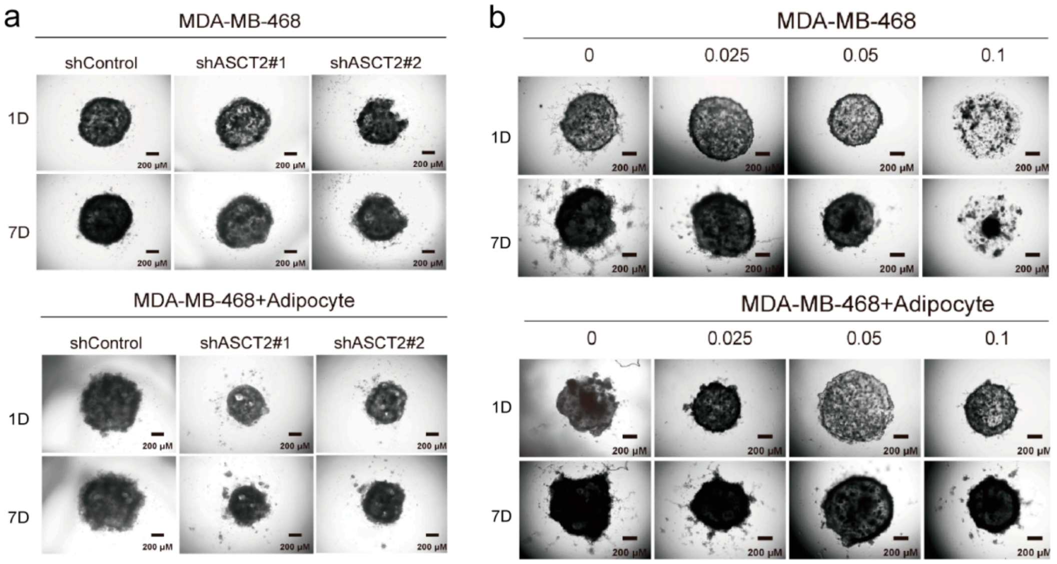

Figure S5: C118P inhibited breast cancer cell proliferation in the co-culture system. (a) ASCT2-knockdown cells were cultured in a 3D system and photos were captured for 7 consecutive days. (b) Breast cancer cells were treated with 0.025, 0.05, or 0.1 μM C118P, cultured in a 3D model, and photos were captured for 7 consecutive days.

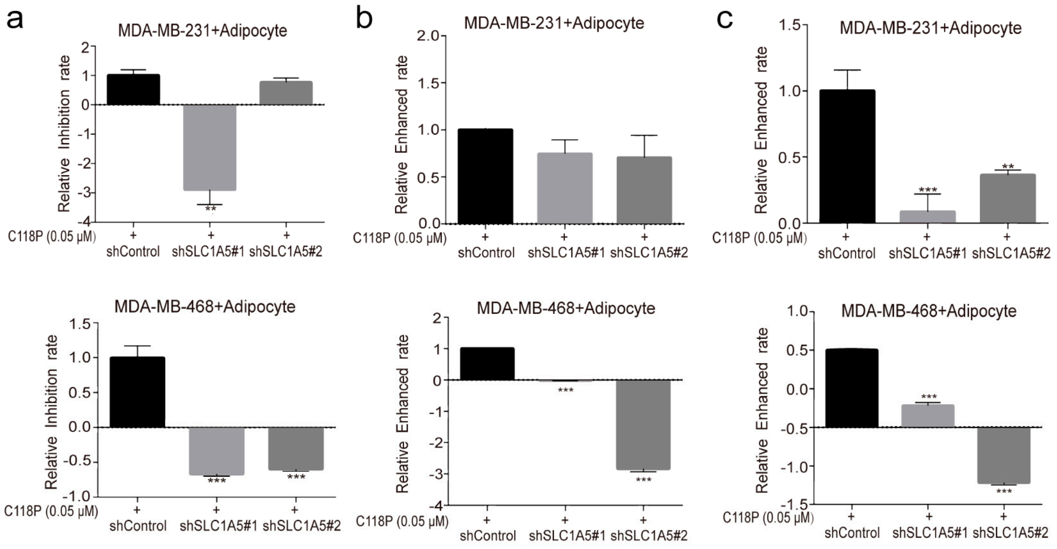

Figure S6: C118P inhibiting cell metabolism of human breast cancer cells in cocluture system. Human breast cancer cells and ASCT2-knockdown cells were co-cultured for three days in the presence or absence of adipocytes. Different doses of C118P were added in the culture medium for 48 h. ATP production (a), glucose uptake (b) and lactate production (c) were detected after treatment with C118P for 48 h in human breast cancer cells. Quantitative Results are representative of three independent experiments mean ± SD. Data are analyzed using the Student's t-test was statistically significant; **P<0.01, ***P<0.001 vs. shControl group.

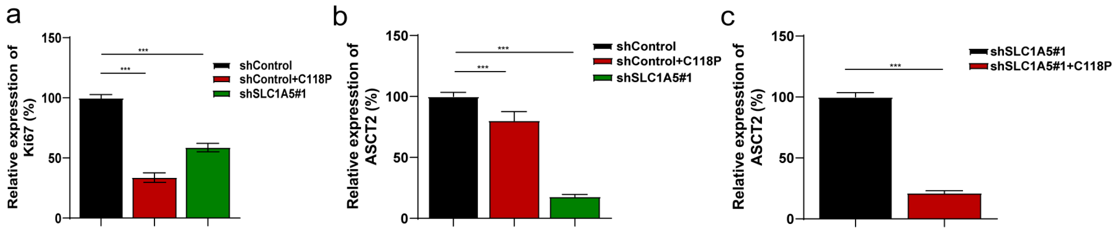

Figure S7: The statistical analysis of Ki67 positive cells and ASCT2 positive cells related to Figure 8. a-c The statistical analysis of Ki67, ASCT2 and Ki67, respectively. Data are analyzed using the Student's t-test was statistically significant; ***P<0.001 vs. shControl group.
