# Supplementary figures and images for: A Novel ASCT2 Inhibitor, C118P, Blocks Glutamine Transport and Exhibits Antitumour Efficacy in Breast Cancer
Source: Cancers (Basel). 2023 Oct 20;15(20):5082. doi: 10.3390/cancers15205082 (PMC10605716; doi:10.3390/cancers15205082)

Fig.1d

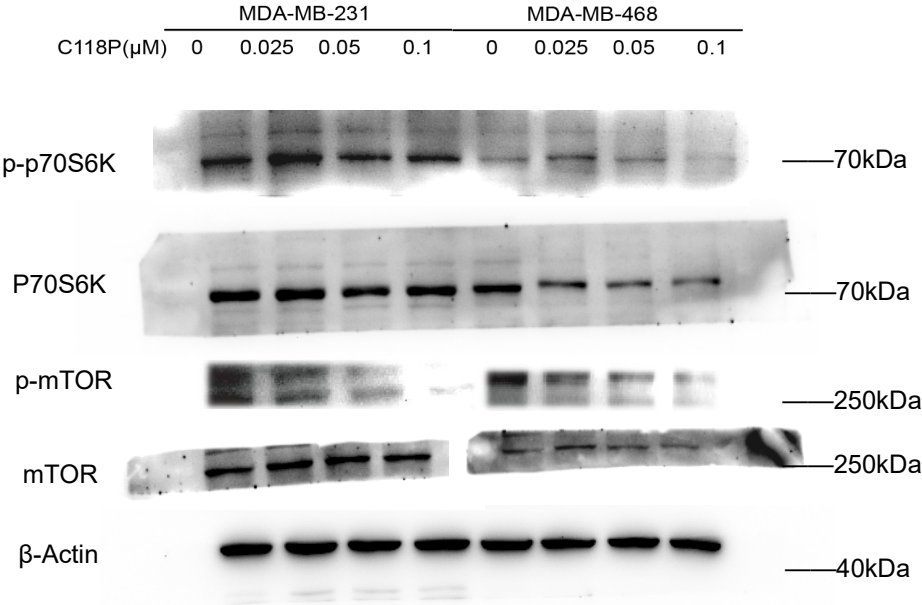

Fig.2e-f

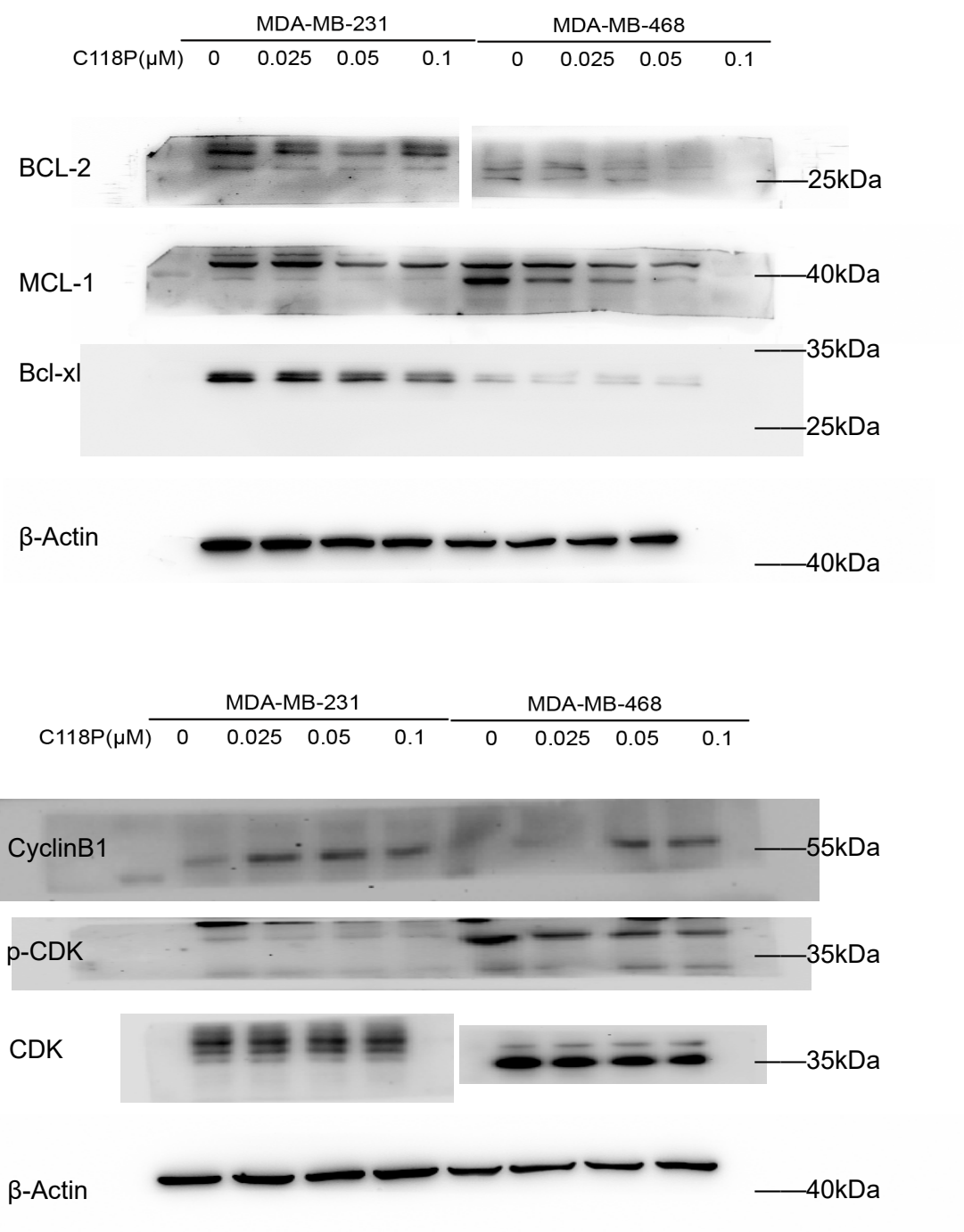

Fig.4d-e

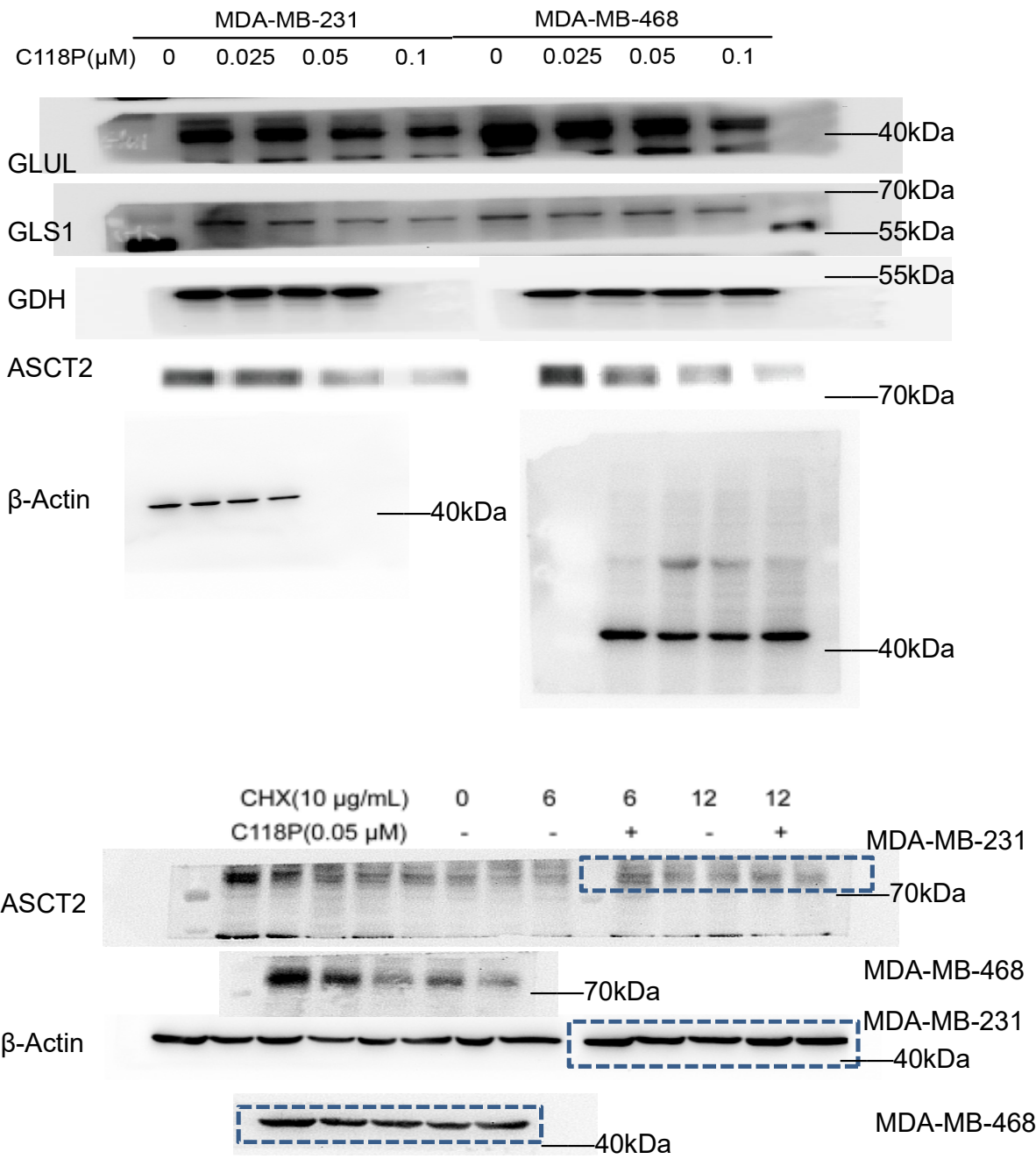

Fig.4f-g

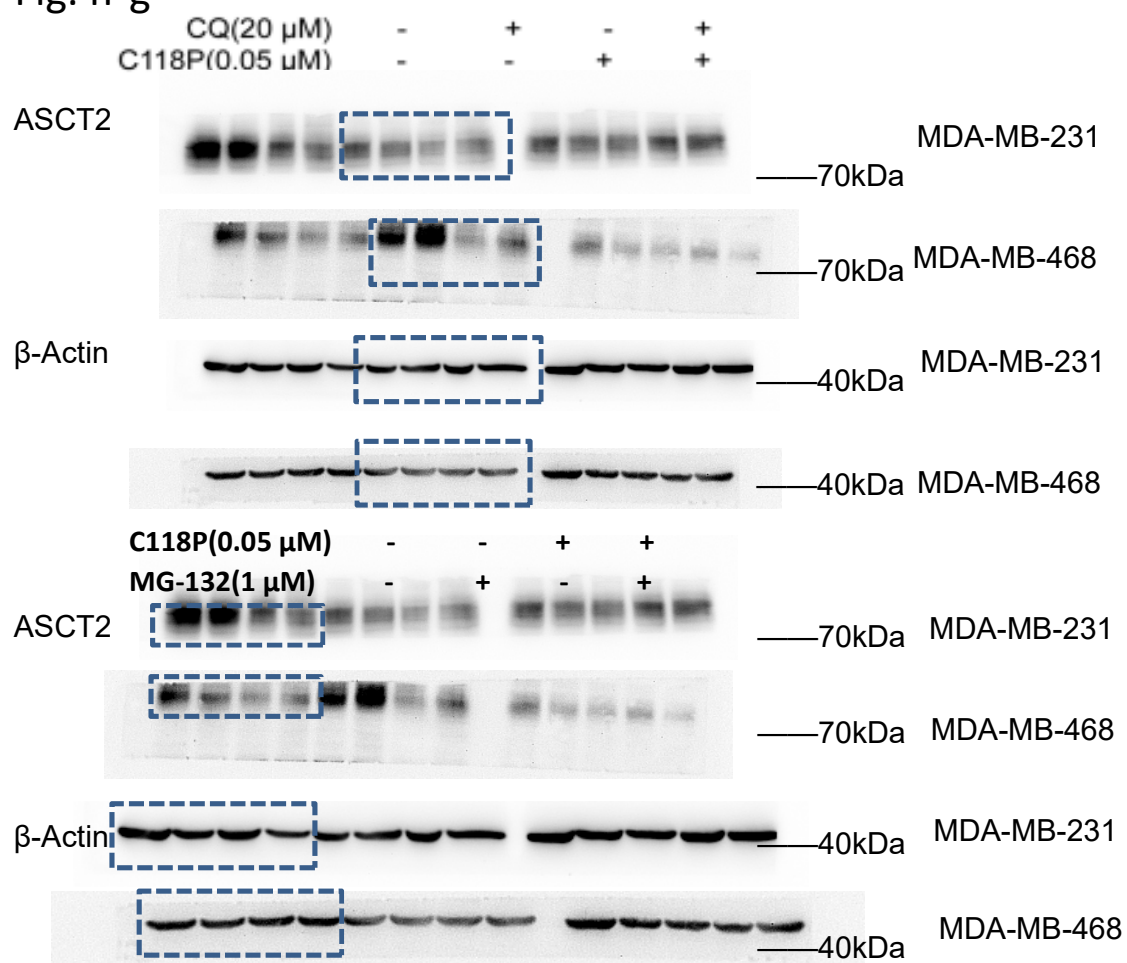

Fig.4h

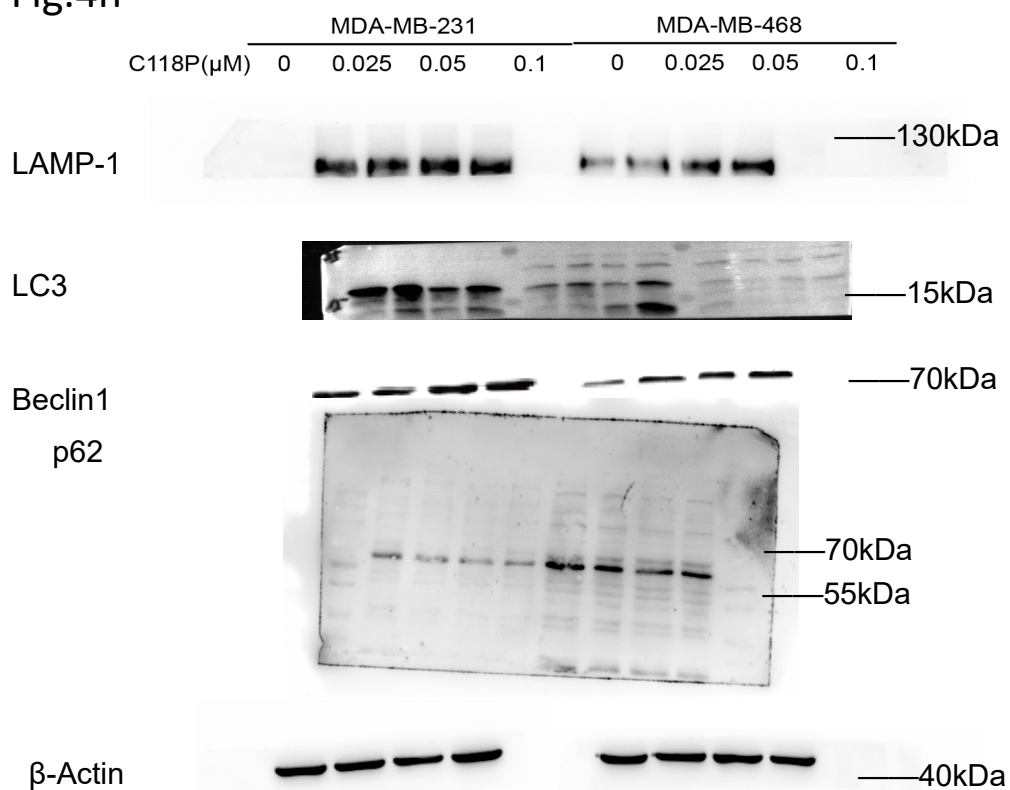

Fig.6b, h

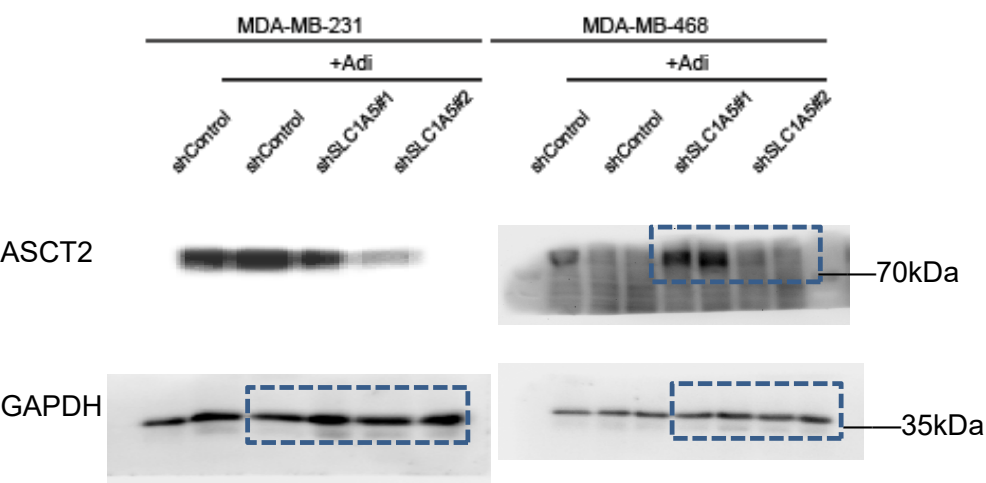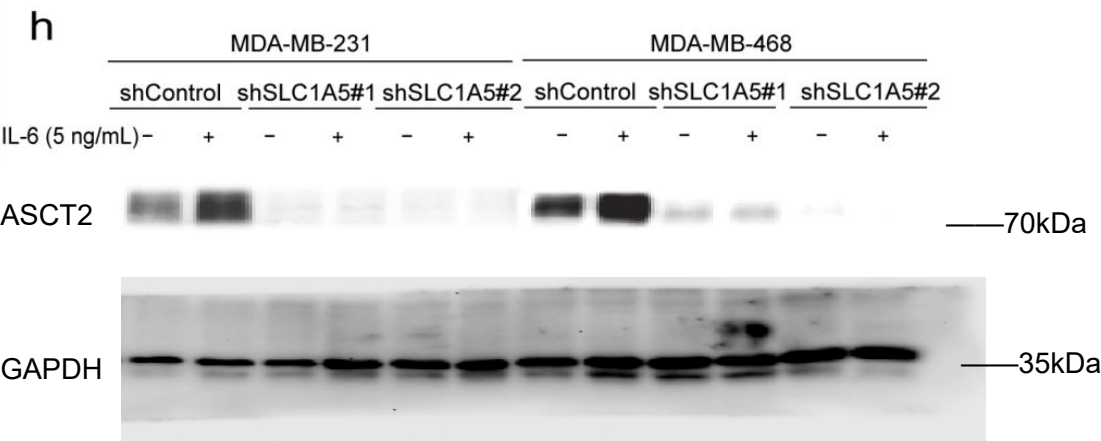

Fig.7f

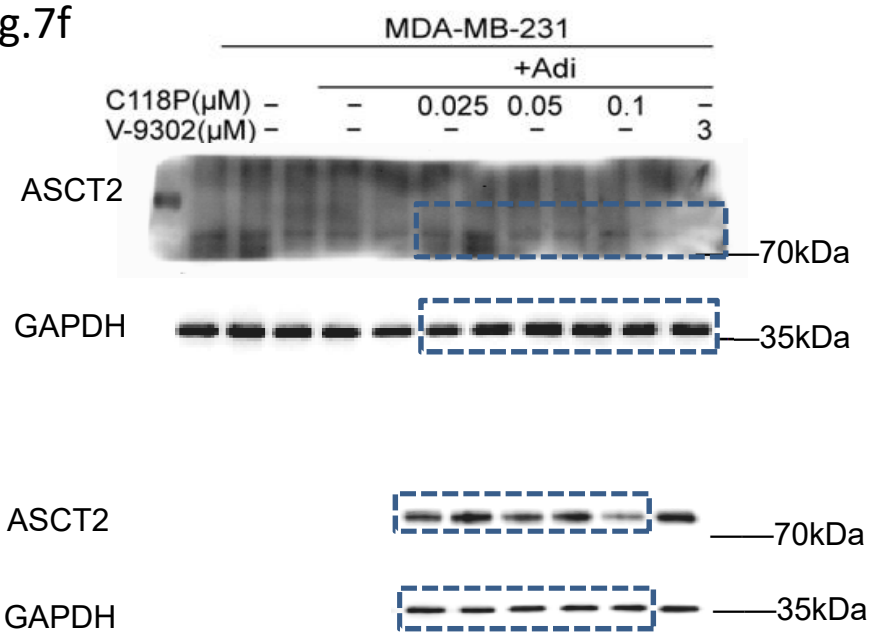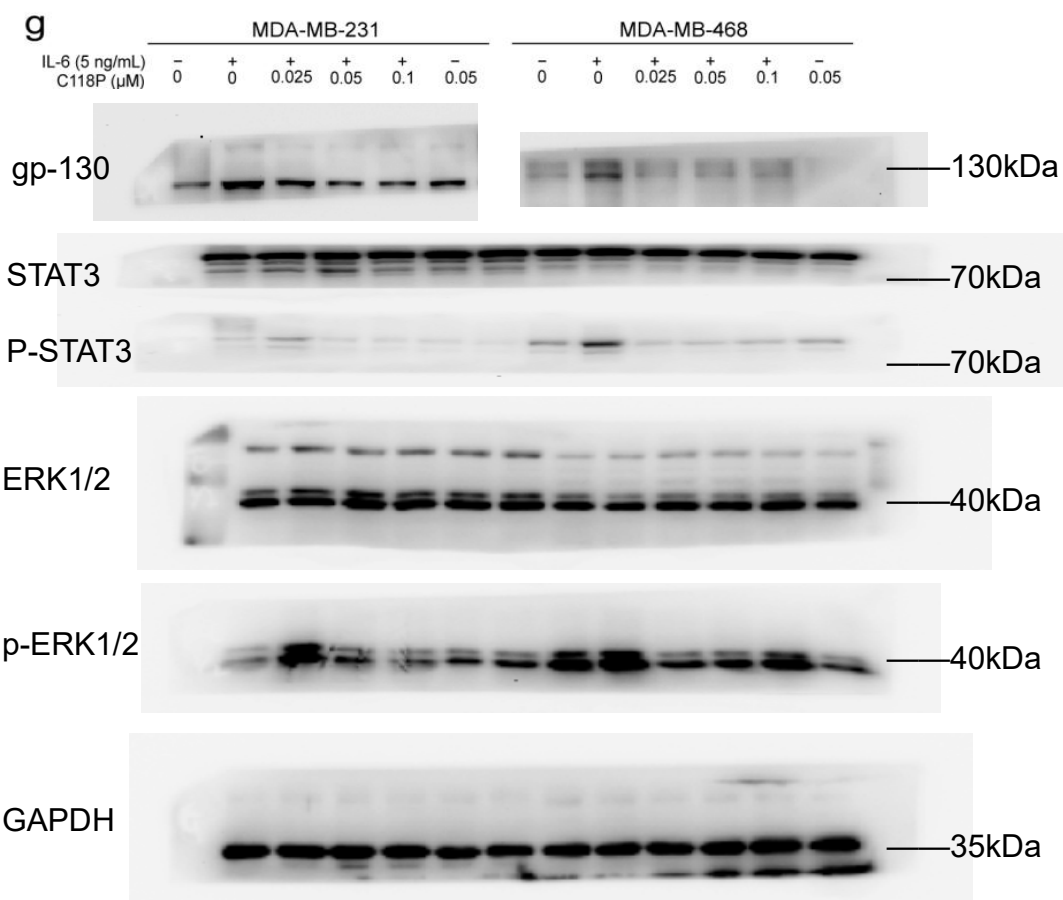

Supplement: Supplementary file 1 [file cancers-15-05082-s001.zip › Supplementary Materials-The uncropped bolts of Wetern blots.pdf]
